# The cellular mechanisms associated with the anesthetic and neuroprotective properties of xenon: a systematic review of the preclinical literature

**DOI:** 10.3389/fnins.2023.1225191

**Published:** 2023-07-14

**Authors:** Steven McGuigan, Daniel J. Marie, Liam J. O'Bryan, Francisco J. Flores, Lisbeth Evered, Brendan Silbert, David A. Scott

**Affiliations:** ^1^Department of Anesthesia and Acute Pain Medicine, St. Vincent's Hospital, Melbourne, VIC, Australia; ^2^Department of Critical Care, University of Melbourne, Melbourne, VIC, Australia; ^3^Picower Institute for Learning and Memory, Massachusetts Institute of Technology, Boston, MA, United States; ^4^Department of Anesthesia, Critical Care, and Pain Medicine, Massachusetts General Hospital and Harvard Medical School, Boston, MA, United States; ^5^Department of Anesthesiology, Weill Cornell Medicine, New York, NY, United States

**Keywords:** xenon, anesthesia, neuroprotection, NMDA receptor, ligand gated channels, cell signal cascade, gene transcription, potassium channel

## Abstract

**Introduction:**

Xenon exhibits significant neuroprotection against a wide range of neurological insults in animal models. However, clinical evidence that xenon improves outcomes in human studies of neurological injury remains elusive. Previous reviews of xenon's method of action have not been performed in a systematic manner. The aim of this review is to provide a comprehensive summary of the evidence underlying the cellular interactions responsible for two phenomena associated with xenon administration: anesthesia and neuroprotection.

**Methods:**

A systematic review of the preclinical literature was carried out according to the PRISMA guidelines and a review protocol was registered with PROSPERO. The review included both *in vitro* models of the central nervous system and mammalian *in vivo* studies. The search was performed on 27th May 2022 in the following databases: Ovid Medline, Ovid Embase, Ovid Emcare, APA PsycInfo, and Web of Science. A risk of bias assessment was performed utilizing the Office of Health Assessment and Translation tool. Given the heterogeneity of the outcome data, a narrative synthesis was performed.

**Results:**

The review identified 69 articles describing 638 individual experiments in which a hypothesis was tested regarding the interaction of xenon with cellular targets including: membrane bound proteins, intracellular signaling cascades and transcription factors. Xenon has both common and subtype specific interactions with ionotropic glutamate receptors. Xenon also influences the release of inhibitory neurotransmitters and influences multiple other ligand gated and non-ligand gated membrane bound proteins. The review identified several intracellular signaling pathways and gene transcription factors that are influenced by xenon administration and might contribute to anesthesia and neuroprotection.

**Discussion:**

The nature of xenon NMDA receptor antagonism, and its range of additional cellular targets, distinguishes it from other NMDA antagonists such as ketamine and nitrous oxide. This is reflected in the distinct behavioral and electrophysiological characteristics of xenon. Xenon influences multiple overlapping cellular processes, both at the cell membrane and within the cell, that promote cell survival. It is hoped that identification of the underlying cellular targets of xenon might aid the development of potential therapeutics for neurological injury and improve the clinical utilization of xenon.

**Systematic review registration:**

https://www.crd.york.ac.uk/prospero/, identifier: 336871.

## 1. Introduction

The monoatomic gas xenon has long been considered a useful medical gas within anesthesia, critical care and in medical imaging. It can be utilized as the primary hypnotic agent for general anesthesia and its use presents several advantages over traditional anesthetic agents that include: greater hemodynamic stability, faster recovery of consciousness, reduced environmental impact and potentially greater neuroprotection (Law et al., 2016; Nair et al., 2021). Xenon has also shown promise as a therapeutic that might improve outcomes following traumatic or hypoxic-ischemic brain injury (Anna et al., 2020; Maze and Laitio, 2020).

Whilst the significant cost of xenon and the specialized apparatus required to deliver and monitor it safely remain a barrier to use, xenon has the potential to provide therapeutic benefits for conditions that have proven difficult to prevent and treat, such as peri-operative neurocognitive disorders, and hypoxic and traumatic brain injuries (Anna et al., 2020; Maze and Laitio, 2020; Nair et al., 2021).

There is significant preclinical evidence that xenon has neuroprotective effects against a range of neurotoxic insults, when utilized pre-, during or post-injury (Van Hese et al., 2018). However, these findings have not, to date, translated to improved cognitive outcomes following xenon anesthesia (Nair et al., 2021). The failure to translate promising cell or animal model findings into clinical benefits is not unique to xenon (Kharasch, 2018). One potential for the failure of translation from bench to bedside is flawed methodology in preclinical *in vivo* and *in vitro* studies, leading to bias and unreliable conclusions (Seyhan, 2019).

The mechanism by which xenon exerts its anesthetic and neuroprotective effects remains uncertain. There is an abundance of literature that details the interaction of xenon with membrane bound proteins, intracellular proteins, second messenger systems and gene transcription. However, the relative importance of these molecular interactions is not clear and previous reviews of xenon's molecular method of action have been narrative rather than systematic (Preckel et al., 2006; Dickinson and Franks, 2010; Winkler et al., 2016).

Whilst the complete outer electron shell of xenon renders it chemically inert, the presence of a large electron cloud also confers xenon with a large degree of polarizability (Roose et al., 2018). It is this property which allows xenon to interact with the amino acid residues of proteins. By occupying hydrophobic pockets within proteins, xenon can rigidify proteins and alter their function (Colloc'h et al., 2007; Roose et al., 2018). Protein crystallography studies have identified xenon binding to commonly investigated analogs of both membrane bound and intracellular globular proteins (Colloc'h et al., 2007; Roose et al., 2018).

The aim of this review is to identify the underlying cellular interactions responsible for two phenomena observed at the whole organism level during xenon administration: (1) anesthesia, including loss of consciousness and the loss of response to noxious stimuli and (2) neuroprotection, the protection of central nervous system (CNS) cells and tissues from injury. The mechanisms underlying these two phenomena are likely to involve significant overlap and therefore the focus of the review is on identifying the cellular targets of xenon relevant to either phenomenon, as opposed to distinguishing which targets are responsible for each. By applying a systematic approach to the literature, we aim to provide a comprehensive summary of the cellular targets of xenon relevant to xenon anesthesia and neuroprotection.

## 2. Methods

### 2.1. Study protocol

The study protocol was developed according to the PRISMA guidelines (Page et al., 2021). Whilst the PRISMA guidelines are primarily designed to summarize aggregate data from clinical studies, the guidelines author's encourage the use of the PRISMA protocol in other research fields when specific systematic review guidance is not available (Page et al., 2021). No standard review framework is available in the basic sciences and the PRISMA guidelines have been recommended for use by authors in the basic science community (O'Hagan et al., 2018; Mikolajewicz and Komarova, 2019). The PROSPERO database was searched prior to development of the study protocol and no studies addressing the research question were identified to be ongoing or recently completed. The study protocol was registered with PROSPERO prior to data extraction.

### 2.2. Eligibility criteria

The PECO (Population, Exposure, Comparator, Outcome) formulation was utilized to develop the eligibility criteria for studies within this review. In recognition of the diversity of studies covered by the review, the PECO framework was applied separately for *in vitro* and *in vivo* studies. The formulation for each is summarized in Table 1.

**Table 1:** PECO formulation.

***In vivo*** **studies**	
Population	All mammalian species
Exposure	Exposure to xenon (all doses, timings, and routes of administration)
Comparator	Control animals not exposed to xenon Control animals exposed to an antagonist for a proposed site of xenon action Wild type animals (when compared to knockout animals)
Outcome	Quantitative data on the expression of proteins or mRNA Behavioral outcomes or injury quantification outcomes in which a target for xenon is identified
***In vitro*** **studies**	
Population	Cell models of neurons Cultured neurons Brain and spinal cord tissue slices Single bouton preparations of neurons
Exposure	Exposure to xenon (all doses, timings, and routes of administration)
Comparator	Cells or tissues not exposed to xenon or following xenon washout Cells or tissue models exposed to an antagonist for a proposed site of xenon action Wild type receptor models (when compared to transgenic receptor models)
Outcome	Electrophysiological measurements of ionic current Quantitative data on the activity of membrane transport proteins Quantitative data on the expression of proteins or mRNA Injury quantification outcomes in which a target for xenon is identified

There were no time or language restrictions on studies eligible for inclusion. To be eligible for inclusion, studies had to have adequate reporting of methods such that the experiments within the study could be reproduced. Exclusion criteria included review articles, conference abstracts and letters that did not provide adequate reporting of methods. The review did not include *in-silico* (computer) simulation studies or x-ray crystallography studies of xenon-protein interactions.

An important distinction in the outcomes eligible for inclusion was made between studies that identified and tested a hypothesis for a proposed site of action for xenon and those that did not. There is a significant literature describing the ability of xenon to prevent CNS cell death and injury in response to a variety of neurotoxic insults. Reviews of this literature are available (Maze, 2016; Van Hese et al., 2018; Anna et al., 2020). The aim of this review was to identify the underlying cellular interactions of xenon that are responsible for anesthesia and neuroprotection. Whilst the review includes several injury quantification studies, each of the included studies tests a specific hypothesis regarding the site of action of xenon.

Another important distinction is made between quantification of proteins and molecules considered potential modulators of neuroprotection, which are included, and those which are regarded simply as markers of cell injury, which are excluded. A specific list of those proteins and molecules considered modulators and those considered markers is included in the Supplementary material.

### 2.3. Search strategy

The search strategy was developed by the research team with additional assistance from a senior librarian familiar with systematic review search development. The search was performed on May 27th, 2022 in the following databases: Ovid Medline, Ovid Embase, Ovid Emcare, APA PsycInfo and Web of Science. The Polyglot search translator (https://sr-accelerator.com/#/polyglot) was utilized to translate search strings. The search terms can be summarized as a combination of “xenon” AND (“anesthesia” OR “neuroprotection”), AND “cellular method of action”. The complete search terms utilized for Ovid Medline are presented in the Supplementary material.

### 2.4. Selection and data collection process

A team of three independent reviewers (SM, DM, LO), including the primary author (SM), screened the title and abstract of all articles identified by the search. Two of the three reviewers, one of whom had to be the primary author (SM), were required to screen each article at this stage. Each study was assessed against the eligibility criteria and full text articles obtained for all articles that were identified as eligible by one or both reviewers in an independent scoring process. The full text articles obtained from this process were reviewed by the primary author (SM) and an independent reviewer (DM, LO) against the eligibility criteria and each reviewer independently identified the articles as either eligible or ineligible. Non-unanimous decisions were resolved through discussion and eligible articles proceeded to data collection.

Data collection was performed utilizing a template designed for the purpose by the primary author. Two members of the research team (SM, DM) independently collected data from the included studies. Discrepancies in data collection were resolved through discussion.

### 2.5. Risk of bias estimation

Standard tools for risk of bias (RoB) estimation such as the SYRCLE risk of bias tool (Hooijmans et al., 2014), and the Cochrane Collaboration risk of bias tool (Higgins et al., 2011) are available for animal studies and clinical studies. In contrast, the use of RoB estimation tools for *in vitro* reviews is inconsistent. A recent systematic review of *in vitro* studies identified that the majority utilized a RoB tool developed by the authors themselves for their specific purpose (Tran et al., 2021).

In this review, we utilized the Office of Health Assessment and Translation (OHAT, National Toxicology Program, US) RoB tool (Eick et al., 2020). This tool has been adapted for the inclusion of both *in vitro* and *in vivo* studies and is on a list of recommended quality assessment tools provided by the National Health and Medical Research Council of Australia (www.NHMRC.org.au). Two independent reviewers (SM, DM) scored the articles according to the OHAT tool and discrepancies in the scoring of articles were resolved through discussion.

## 3. Results

### 3.1. Study selection

**Figure 1:**
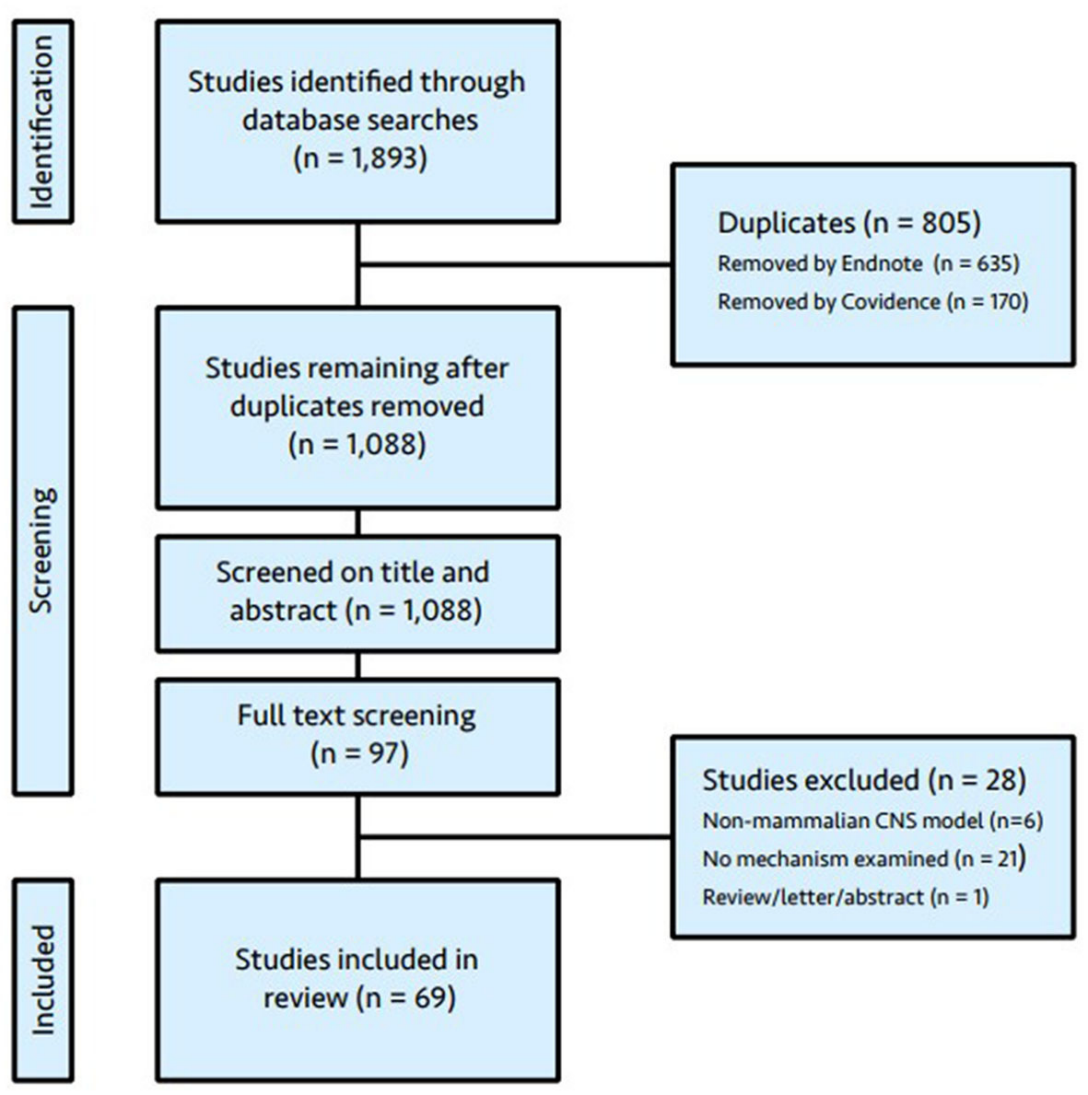
PRISMA flow diagram.

The literature search yielded 1893 articles. Duplicates were removed utilizing automated identification in EndNote X9.2 (Clarivate, Chandler, AZ, USA) and Covidence systematic review software (Veritas Health Innovation, Melbourne, Australia), followed by manual deletion by a member of the research team (LO). Following duplicate removal, 1,088 articles were available for title and abstract screening. Of these, 97 were considered eligible by at least one reviewer and the full text retrieved. The full text was available for all articles. Twenty-eight articles were excluded following review and the reasons for exclusion are given in Figure 1 (PRISMA flow diagram). No further articles were identified by cross-referencing. The results from a letter to the editor (Kratzer et al., 2017), which outlined retractions and a repeat experiment from one of the eligible studies (Mattusch et al., 2015), were incorporated with the original article.

### 3.2. Study characteristics

Of the 69 included articles, xenon was administered to live animals (*in vivo*) in 21 studies and to tissues and cell cultures (*in vitro*) in 46 studies. Three studies utilized both *in vivo* and *in vitro* methods. One of these studies was the subject of a letter which retracted the results of the *in vivo* experiment (Mattusch et al., 2015). The results of that *in vivo* experiment were excluded from the data synthesis leaving twenty-three studies that included at least one *in vivo* experiment in the analysis.

The characteristics of studies that included an *in vivo* experiment are given in Table 2. The majority of studies were performed in rats. Of those studies that provided the sex of the animal, most were performed in male animals. Most studies did not report the sex of the animals utilized. Xenon was delivered as an inhaled gas in most studies although some studies did utilize injection of xenon with a lipid carrier. The most common outcome reported for *in vivo* studies was quantification of proteins or messenger RNA (mRNA).

**Table 2:** Study characteristics.

**Category**	**Number of studies**
**Xenon exposure**	
*In vivo*	21
*In vitro*	46
Both	2
***In vitro*** **studies**	
**Sex**	
Male	10
Female	0
Both	1
Not identified	12
**Age**	
Young	9
Adult	8
Not specified	6
**Species**	
Rat	20
Mouse	3
**Xenon administration**	
Inhalational	19
Intravenous	3
Other	1
**Outcome**	
Protein quantification	15
mRNA quantification	3
Both	3
MAC	1
Membrane transport	1
***In vitro*** **studies**	
**Cell/tissue model**	
Cultured cell	31
Tissue slice	13
Both	2
Single bouton	2
**Outcome**	
Membrane transport	39
Injury quantification	5
Protein quantification	1
Multiple	3

The characteristics of those studies that included an *in vitro* experiment are also given in Table 2. The majority of studies utilized cultures of either native or transgenic cells, although tissue slices and the single bouton technique were also utilized. The most common outcome from *in vitro* studies was the quantification of membrane bound protein activity, either ionic current transfer or transfer of other molecules.

### 3.3. Risk of bias assessment

Risk of bias responses were recorded for all nine domains of the OHAT risk of bias assessment for studies that had at least one *in vivo* experiment (Supplementary Table 1). For the remaining *in vitro* studies, the responses for randomization and allocation concealment were omitted, as per the OHAT Handbook, leaving seven responses to record. Only one study recorded a “definitely low” risk of bias across all responses. No studies recorded a “definitely high” response.

Fifty-five studies recorded at least one “probably high” response (80.0%). The “exposure characterization” domain recorded the most “probably high” responses (39.1%). This represents studies which did not independently verify the concentration of xenon during experiments. The matrix of responses for each study and a summary of all responses is provided in the Supplementary material.

## 4. Results of individual studies

Given the heterogeneity of study designs and study outcomes included in the review, the results are presented as a narrative synthesis. The included studies described 638 individual experiments in which a hypothesis was tested regarding the effect of xenon exposure on a specific cellular target. The results of individual studies are divided into three sections based on the cellular target influenced by xenon: membrane bound proteins (Section 4.1), intracellular signaling pathways (Section 4.2) and gene transcription (Section 4.3).

The results of studies that investigated more than one cellular target may be presented in more than one section. The results of individual studies are also presented in tables summarizing the results of three outcomes: membrane bound protein activity (Tables 3–**5**), protein and mRNA quantification (**Tables 6**, **7**), and injury quantification (**Table 8**).

**Table 3:** Summary of membrane bound proteins—ionotropic glutamate results.

**References**	**Model**	**Cell line/tissue**	**Species (receptor)**	**Receptor subtype**	**Xenon dose**	**Outcome reported**	**Direction of effect**
**NMDA**							
David et al. (2003)	Cultured cells	Neuronal/glial co-culture	Mouse		75%	Intracellular calcium	**↑** **↓** ^*^
Weigt et al. (2003)	Cultured cells	Cerebral cortex	Mouse		Various	Ionic current	**↓**
David et al. (2003)	Cultured cells	Neuronal/glial co-culture	Mouse		50%	Intracellular calcium	**↓**
Ogata et al. (2006)	Cultured cells	Xenopus oocytes	Human	NR1-NR2A	46%	Ionic current	**↓**
Solt et al. (2006)	Cultured cells	Xenopus oocytes	Human	NR1-NR2B	70%	Ionic current	**↓**
Dickinson et al. (2007)	Cultured cells	HEK293	Rat	NR1-NR2A/NR2B	80%	Ionic current	**↓**
Weigt et al. (2008)	Cultured cells	Neuro2A	Rat/mouse	NR1-NR2A/B/C/D	84%	Ionic current	**↓**
Weigt et al. (2009a)	Cultured cells	Neuro2A	Unknown	NR1-NR2A	84%	Ionic current	**↓**
Weigt et al. (2009b)	Cultured cells	Cerebral cortex	Mouse		Various	Ionic current	**↓**
Yamakura and Harris (2000)	Cultured cells	Xenopus oocytes	Human	NR1-NR2A	46%	Ionic current	**↓**
Armstrong et al. (2012)	Cultured cells	HEK293	Rat	NR1-2A	80%	Ionic current	**↓**
Haseneder et al. (2008)	Tissue slice	Amygdala	Mouse		65%	Ionic current	**↓**
Haseneder et al. (2008)	Tissue slice	Amygdala	Mouse		30%	Ionic current	**↓**
Haseneder et al. (2009a)	Tissue slice	Amygdala	Mouse		65%	Ionic current	**↓**
Haseneder et al. (2009b)	Tissue slice	Prefrontal cortex	Mouse		65%	Ionic current	**↓**
Haseneder et al. (2009b)	Tissue slice	Dorsal spinal cord	Mouse		65%	Ionic current	**↓**
Haseneder et al. (2009b)	Tissue slice	Prefrontal cortex	Mouse		30%	Ionic current	**↓**
Georgiev et al. (2010)	Tissue slice	Dorsal spinal cord	Rat		50%	Ionic current	**↓**
Kratzer et al. (2012)	Tissue slice	Hippocampus	Mouse		65%	Ionic current	**↓**
Yamamoto et al. (2012)	Tissue slice	Ventral spinal cord	Rat		50%	Ionic current	**↔**
Baufreton et al. (2018)	Tissue slice	Cortico-striatal	Mouse		50%	NMDA/AMPA ratio	**↓**
Nonaka et al. (2019)	Single bouton	Hippocampal CA3 neuron	Rat		70%	Ionic current	**↓**
Kubota et al. (2020)	Single bouton	SDCN	Rat		70%	Ionic current	**↓**
**AMPA**							
Plested et al. (2004)	Cultured cells	Xenopus oocyte	Rat	Homomeric	80%	Ionic current	**↑** **↔** ^*^
Plested et al. (2004)	Cultured cells	Xenopus oocyte	Rat	Heteromeric	80%	Ionic current	**↔**
Dinse et al. (2005)	Cultured cells	Cortical neurones	Mouse		84%	Ionic current	**↓**
Weigt et al. (2009a)	Cultured cells	Neuro2A	Unknown	Homomeric	84%	Ionic current	**↓**
Haseneder et al. (2008)	Tissue slice	Basolateral amygdala	Mouse		65%	Ionic current	**↓**
Haseneder et al. (2009b)	Tissue slice	Prefrontal cortex	Mouse		65%	Ionic current	**↓**
Haseneder et al. (2009b)	Tissue slice	Dorsal spinal cord	Mouse		65%	Ionic current	**↓**
Haseneder et al. (2009b)	Tissue slice	Prefrontal cortex	Mouse		30%	Ionic current	**↓**
Georgiev et al. (2010)	Tissue slice	Dorsal spinal cord	Rat		50%	Ionic current	**↓**
Kratzer et al. (2012)	Tissue slice	Hippocampal brain slice	Mouse		65%	Ionic current	**↓**
Yamamoto et al. (2012)	Tissue slice	Ventral spinal cord	Rat		50%	Ionic current	**↓**
Yamamoto et al. (2012)	Tissue slice	Ventral spinal cord	Rat		75%	Ionic current	**↓**
Nonaka et al. (2019)	Single bouton	Hippocampal CA3 neurone	Rat		70%	Ionic current	**↓**
Kubota et al. (2020)	Single bouton	SDCN	Rat		70%	Ionic current	**↓**
**Kainate**							
Dinse et al. (2005)	Cultured cells	Cortical neurones	Mouse		84%	Ionic current	**↓**
Dinse et al. (2005)	Cultured cells	SH-SY5Y	Rat	GluR6	84%	Ionic current	**↓**
Nonaka et al. (2019)	Single bouton	Hippocampal CA3 neuron	Rat		70%	Ionic current	**↓**
Kubota et al. (2020)	Single bouton	SDCN	Rat		70%	Ionic current	**↓**
**Glutamate (receptor non-specified)**							
de Sousa et al. (2000)	Cultured cells	Hippocampal neurones	Rat		80%	Charge transfer	**↓**
de Sousa et al. (2000)	Cultured cells	Hippocampal neurones	Rat		80%	Slow component	**↓**
de Sousa et al. (2000)	Cultured cells	Hippocampal neurones	Rat		80%	Fast component	**↔**
Haseneder et al. (2008)	Tissue slice	Amygdala	Mouse		65%	Ionic current	**↓**
Haseneder et al. (2009b)	Tissue slice	Prefrontal cortex	Mouse		65%	Ionic current	**↓**
Haseneder et al. (2009b)	Tissue slice	Dorsal spinal cord	Mouse		65%	Ionic current	**↓**
Haseneder et al. (2009b)	Tissue slice	Prefrontal cortex	Mouse		65%	Postsynaptic transmission	**↓**
Haseneder et al. (2009b)	Tissue slice	Prefrontal cortex	Mouse		65%	Presynpatic transmission	**↔**
Haseneder et al. (2009b)	Tissue slice	Dorsal spinal cord	Mouse		65%	Postsynaptic transmission	**↓**
Haseneder et al. (2009b)	Tissue slice	Dorsal spinal cord	Mouse		65%	Presynpatic transmission	**↔**
Yamamoto et al. (2012)	Tissue slice	Ventral spinal cord	Rat		50%	Postsynaptic transmission	**↓**
Yamamoto et al. (2012)	Tissue slice	Ventral spinal cord	Rat		50%	Presynpatic transmission	**↔**
Nonaka et al. (2019)	Single bouton	Hippocampal CA3 neuron	Rat		70%	Presynpatic transmission	**↓**
Nonaka et al. (2019)	Single bouton	Hippocampal CA3 neuron	Rat		70%	Postsynaptic transmission	**↓**
Nonaka et al. (2019)	Single bouton	Hippocampal CA3 neuron	Rat		30%	Presynpatic transmission	**↓**
Nonaka et al. (2019)	Single bouton	Hippocampal CA3 neuron	Rat		30%	Postsynaptic transmission	**↓**
Kubota et al. (2020)	Single bouton	SDCN	Rat		70%	Presynaptic transmission	**↓**
Kubota et al. (2020)	Single bouton	SDCN	Rat		70%	Postsynaptic transmission	**↓**

Direction of effect: ↑–Increased, ↓–Reduced, ↔–No change.

Color of arrow represents statistics presented by authors: Green—*p*-value in comparison to control given, Amber—no *p*-value but standard error of mean presented for xenon and control groups, Red—*p*-value/SEM not given for xenon and control groups.

Presynaptic transmission include measures of miniature, spontaneous and evoked potentials that indicate presynaptic mechanisms. Postsynaptic transmission include miniature, spontaneous and evoked potentials that indicate postsynaptic mechanisms. Details given within text.

SDCN, Sacral dorsal commissural nucleus.

* Dependent on experimental conditions, see text for details.

The concentration of xenon applied during experiments was not described in a uniform manner by authors. In some instances, the concentration of xenon gas allowed to come to equilibrium with a test solution was provided. In other instances, the concentration of xenon within the solution itself in millimoles (calculated utilizing solubility coefficients or measured directly by gas chromatography) was provided. To provide comparable results, the tables provide the concentration of xenon gas either applied directly to tissues (e.g., in a gas chamber), or the concentration of gas that, at equilibrium, would produce a test solution of the concentration reported. In the case of xenon delivered in a lipid carrier, the dose of lipid solution (in mg^−1^ kg^−1^) is provided.

### 4.1. Membrane bound proteins

#### 4.1.1. Ionotropic glutamate receptors

##### 4.1.1.1. NMDA receptor

The N-methyl-D-aspartate receptor (NMDAR) is one of the principal targets of glutamate, the most abundant excitatory neurotransmitter of the CNS. The NMDAR is reportedly responsible for the anesthetic actions of agents such as ketamine and nitrous oxide (Akeju et al., 2016), and in the propagation of CNS injury (Hansen et al., 2017).

The vast majority of electrophysiology experiments within the review identified that xenon exposure reduced the ionic current through the NMDAR when the receptor was activated by either pharmacological or electrical methods. Reduced ionic current was identified in all models of neurons including murine cell cultures, transfected cells, single bouton preparations and tissue slices.

In one study of the ventral spinal cord, xenon did not alter ionic current through the NMDAR (Yamamoto et al., 2012). This study reported the effect of xenon on electrically and pharmacologically evoked excitatory postsynaptic currents (EPSC) in lamina IX neurons in a tissue slice preparation of rat lumbosacral spinal cord. Xenon at 50% did not affect the electrically evoked response, and xenon at 50 or 75% did not affect the pharmacologically evoked response.

Two other studies investigated the effects of xenon in tissue slice preparations of dorsal murine spinal cord neurons. One study measured amplitude and area of EPSC evoked by the application of NMDA (Georgiev et al., 2010) and the other measured relative amplitude of the EPSC evoked by both pharmacological and electrical activation (Haseneder et al., 2009a). Both studies identified a reduction in current amplitude to around 60% of control responses.

Whilst variable responses to xenon at different anatomical sites within the spinal cord were reported, the results of tissue slice studies of the brain were more uniform. Studies of the amygdala, prefrontal cortex, hippocampus, and cortico-striatal pathways all identified reductions in ionic current through NMDAR in the presence of xenon (Haseneder et al., 2008, 2009a,b; Kratzer et al., 2012; Baufreton et al., 2018).

One study (Dickinson et al., 2007) performed a direct comparison of the effect of xenon on ionic current through NR1-NR2A and NR1-NR2B receptors and identified no significant difference in xenon inhibition between them. Another study reported that xenon exposure resulted in inhibition of NMDAR assembled with each of the four NR2 subtypes (NR2A-D) (Weigt et al., 2008).

One study investigated the effect of varying concentrations (5 to 65%) of xenon on electrically and pharmacologically evoked EPSC in murine basolateral amygdala (Haseneder et al., 2008). The reduction in current amplitude was dose dependent and concentrations as low as 18% resulted in a statistically significant reduction in current amplitude. Xenon at 5% did not produce a significant reduction in current. Using similar methodology, the same authors also identified a greater reduction in evoked EPSC in murine prefrontal cortex cells in the presence of 65% xenon when compared to 30% xenon (Haseneder et al., 2009a).

Interestingly, both papers reported no statistical difference in the reductions in current amplitude evoked by pharmacological or electrical means. From this, the authors concluded that xenon's ability to inhibit NMDAR ionic current is likely a postsynaptic phenomenon since their model of photolytic release of caged L-glutamate was designed for specific activation of postsynaptic receptors (Haseneder et al., 2008, 2009a).

The effect of concentration was also investigated in a neuronal/glial co-culture (David et al., 2003). The authors utilized video fluoromicroscopy to measure calcium influx stimulated by varying concentrations of NMDA, in the presence and absence of 50 and 75% xenon. Whilst 50% xenon significantly reduced calcium influx at all concentrations of NMDA, 75% xenon had a “bivalent” effect, reducing influx at concentrations below 25 μM NMDA but potentiating the increase at 50–100 μM.

This “bivalent” effect is in contrast to the electrophysiology literature in which studies at this concentration, and above, all reported significant reductions in ionic current (Dickinson et al., 2007; Weigt et al., 2008, 2009a; Armstrong et al., 2012). Two studies that delivered xenon in a lipid emulsion also suggest that inhibition of NMDAR is dose-dependent (Weigt et al., 2003, 2009b).

Activation of the NMDAR requires a number of conditions which include the binding of both glutamate and glycine (Furukawa et al., 2005). The relationship between glycine concentration and xenon's effect on NMDAR ionic current was investigated by two studies. In both studies, the ability of xenon to reduce ionic current was competitive with, and inversely proportional to, glycine concentration (Dickinson et al., 2007; Armstrong et al., 2012). The effect of 70% xenon on whole cell currents measured at differing concentrations of NMDA reported that xenon non-competitively inhibited ionic current, as the EC50 in the presence and absence of xenon was not significantly different (Nonaka et al., 2019; Kubota et al., 2020).

Several authors explored the effect of point mutations of the NMDAR on xenon inhibition. Two of these studies identified point mutations in the NR1 subunit that reduced xenon inhibition (Ogata et al., 2006; Dickinson et al., 2007). Both mutations were found to increase glycine affinity for the receptor, supporting the hypothesis that xenon's effect is related to competition with glycine. A third study identified NR1 point mutations of the glycine binding site that resulted in reduced xenon inhibition without affecting glycine affinity (Armstrong et al., 2012).

One study investigated the effect of point mutations that altered desensitization of NMDAR (Weigt et al., 2009a). Mutations which greatly diminished or accelerated NMDAR desensitization had no effect on the ability of xenon to inhibit ionic current when compared to wild type NMDAR.

In an attempt to differentiate the action of xenon from open-channel blockers such as magnesium and MK-801, one study (Weigt et al., 2008) identified a point mutation that, after pharmacological manipulation, maintained the NMDAR in a constitutively open state. The authors reported that xenon did not alter the ionic current through such a channel, whilst magnesium and MK-801 maintained their ability to inhibit ionic current.

One study ascertained the minimum alveolar concentration (MAC) to prevent movement in male rats in response to electrical stimulation of the tail in the absence and presence of an infusion of an NMDAR antagonist (MK-801) (Eger et al., 2006). The ability of general anesthetic agents to obtund this response is generally attributed to action at the spinal cord level. The authors reported that the MAC of xenon was maximally reduced to around 60%, when compared to control conditions, by an infusion of 32 μg kg^−1^ min^−1^ of MK-801. Although no formal statistics were performed comparing the groups, the average reduction for all inhaled anesthetics, including the volatiles isoflurane, sevoflurane, enflurane, and halothane was ~60%.

In tissue slice studies, treatment with 50% xenon significantly reduced injury, in the form of oxygen-glucose deprivation or focal trauma, quantified by propidium iodide fluorescence. In all three studies, the co-application of glycine with xenon resulted in an injury that was comparable with untreated controls (Banks et al., 2010; Harris et al., 2013; Koziakova et al., 2019).

In a study of cortical cell cultures, low-level excitotoxicity was modeled by applying pyrrolidine-2,4-dicarboxylate (PDC), a glutamate transport inhibitor, to the culture for four days (Lavaur et al., 2016). Cultures housed in a gas chamber containing 75% xenon (balanced with oxygen), during this period had a greater rate of survival than those housed with 75% nitrogen (balanced with oxygen). Interestingly, in this study, the addition of glycine did not alter the survival rate of neurons when compared to the xenon only treatment group.

##### 4.1.1.2. AMPA receptors

The α-amino-3-hydroxy-5-methyl-4-isoxazolepropionic acid receptor (AMPAR) is an ionotropic glutamate receptor responsible for fast excitatory transmission in the CNS. AMPAR activation also facilitates the activation of NMDAR (Bissen et al., 2019).

In all studies of native cell culture, single bouton and tissue slice preparation, xenon was found to reduce ionic current through AMPAR when compared to control conditions. In these studies, the concentration of xenon ranged from 30 to 84%. Three studies reported the EC50 of agonist in the presence and absence of xenon (Dinse et al., 2005; Nonaka et al., 2019; Kubota et al., 2020). Each reported no statistically significant change in EC50 and concluded that the nature of antagonism was non-competitive.

Xenon reduced AMPA ionic current in single bouton preparations from both the hippocampus and spinal cord dorsal commissural nucleus (Nonaka et al., 2019; Kubota et al., 2020). A reduction in ionic current was also consistent across tissue slice preparations from prefrontal cortex (Haseneder et al., 2009a), amygdala (Haseneder et al., 2008), and lumbosacral dorsal and ventral spinal cord (Haseneder et al., 2009a; Yamamoto et al., 2012).

In one study xenon was applied to a lumbosacral spinal cord segment *in vivo* following laminectomy under urethane anesthesia (Georgiev et al., 2010). The current responses to noxious and non-noxious limb stimuli at the dorsal horn were measured using a voltage clamp technique in the presence of pharmacological NMDAR blockade. The EPSC associated with both touch and pinch stimulation were significantly reduced on exposure of the lumbosacral segment to 50% xenon.

Two studies compared the effect of two different concentrations of xenon. One study identified a greater reduction in ionic current in the prefrontal cortex in the presence of 65% xenon when compared to 30% xenon (Haseneder et al., 2009a). In the other study, of ventral spinal cord neurons, the authors identified a similar significant reduction in ionic current in the presence of 50 and 75% xenon, although no formal comparison of the two concentrations was performed (Yamamoto et al., 2012).

One study also investigated miniature EPSC and the paired pulse ratio in the presence of NMDA blockade in both substantial gelatinosa and prefrontal cortex tissue slices (Haseneder et al., 2009a). Whilst miniature EPSC were reduced in amplitude, the frequency was unchanged in the presence of xenon. The authors also reported no change in paired pulse ratio in the presence of xenon. The authors concluded that these findings, in conjunction with the similar results obtained from pharmacological and electrical stimulation in their experiments, suggest that xenon's action at AMPAR is likely postsynaptic in nature (Haseneder et al., 2009a).

The effect of xenon on AMPAR of differing subunit composition was investigated in transfected cell studies. One study identified significant reductions in ionic current through homomeric GluR1, GLuR3 and GLuR4 AMPAR in response to kainite and glutamate in the presence of desensitization blockade (Plested et al., 2004). However, for heteromeric subtypes the authors identified that only the GluR1-GluR2 subtype was sensitive to xenon. The authors also reported that in a rapid glutamate application system, designed to imitate synaptic conditions, homomeric GluR1 and GluR4 subtypes were relatively insensitive to xenon (Plested et al., 2004).

The effect of point mutations that reduced AMPAR desensitization was reported by two studies (Plested et al., 2004; Weigt et al., 2009a). One study examined the effect of point mutations which reduced AMPAR desensitization in both GluR1 and GluR2 homomeric receptors. The authors reported that the ability of xenon to inhibit ionic current in these AMPAR was significantly reduced, hypothesizing that the sensitivity of AMPAR to xenon is associated with receptor desensitization. This is consistent with an earlier study which identified a non-desensitizing mutant of AMPAR which was also insensitive to xenon (Plested et al., 2004).

##### 4.1.1.3. Kainate receptors

Kainate receptors (KAR) are a key facilitator of synaptic transmission capable of both inhibition and facilitation of excitatory and inhibitory transmission (Contractor et al., 2011). KAR activation is also implicated in glutamate-induced neurotoxicity (Srivastava et al., 2020).

In three studies of native cells, from cortex, hippocampus and spinal cord, 70 and 84% xenon reduced ionic current in response to bath application of kainate when compared to control conditions (Dinse et al., 2005; Nonaka et al., 2019; Kubota et al., 2020). One study reported that the magnitude of this reduction was 42% at the control EC50 of kainate. Two of the studies determined that the nature of the blockade was non-competitive (Dinse et al., 2005; Nonaka et al., 2019), whilst the third was inconclusive (Kubota et al., 2020).

It is well recognized that application of kainate activates AMPAR in addition to kainate-specific receptors (Plested et al., 2004; Dinse et al., 2005). To address this potential confounder, one study utilized a transfected cell line expressing the murine GLuR6 receptor which is specific in its sensitivity to kainate. They reported a reduction in ionic current in response to 84% xenon with no change in the EC50 of kainate, suggesting non-competitive blockade.

##### 4.1.1.4. Glutamatergic excitatory receptors (non-specified)

One study described the effect of xenon on the EPSC recorded in murine autaptic hippocampal neurones (de Sousa et al., 2000). The authors reported a number of features of the EPSC in the presence and absence of xenon including the current peak, total charge transfer and the “slow” and “fast” components of the EPSC. They reported that whilst the peak, total charge transfer and “slow” component were significantly reduced in the presence of xenon, the “fast” component remained unchanged. This led the authors to conclude that the observed effects were likely due to inhibition of NMDAR, rather than AMPAR. This contrasts with multiple subsequent studies discussed in Section 4.1.1.2 that characterized the subtypes of glutamate receptor by utilizing channel specific transfection of cells and pharmacological isolation of NMDAR and AMPAR ionic currents.

Several studies reported outcomes such as miniature EPSC and spontaneous EPSC, and the failure rate and paired pulse ratio of evoked potentials. The study designs and outcomes of these studies were intended to identify if xenon's actions at glutamatergic synapses can be considered presynaptic, postsynaptic or a combination of both.

Three studies investigated the effect of xenon on miniature EPSC in tissue slices of basolateral amygdala as well as the dorsal and ventral spinal cord (Haseneder et al., 2008; Georgiev et al., 2010; Yamamoto et al., 2012). Each study was performed in the presence of tetrodotoxin, to block sodium channels and prevent action potential related release of neurotransmitter, and the concentration of xenon in these studies was either 50 or 65%. Each of these three studies reported that in the presence of xenon the amplitude of miniature EPSC was reduced whilst the frequency remained unchanged. These studies suggest that xenon acts predominantly at the postsynaptic terminal. A fourth study reported similar findings in conditions in which AMPAR miniature EPSC were pharmacologically isolated (Haseneder et al., 2009a).

One study investigated the effect of xenon on miniature EPSC in hippocampal CA3 neurones utilizing the single bouton technique (Nonaka et al., 2019). In contrast with the results from tissue slice studies, this study reported that xenon at a concentration of 70% significantly reduced the frequency of miniature EPSC whilst the amplitude remained unchanged. This study also reported the effect of xenon on amplitude and frequency of spontaneous EPSC. The authors reported that xenon at 30 and 70% reduced frequency but not amplitude of spontaneous EPSC, consistent with a presynaptic mechanism of action.

In a single bouton preparation of sacral dorsal commissural neurones, another study reported that 70% xenon reduced both the frequency and amplitude of spontaneous EPSC (Kubota et al., 2020). Both studies of the single bouton preparation also reported the effects of xenon on a paired pulse electrical stimulus (Nonaka et al., 2019; Kubota et al., 2020). The studies reported that xenon increased the failure rate and increased the paired pulse ratio of evoked EPSC, suggesting the involvement of presynaptic mechanisms.

#### 4.1.2. Other ligand gated membrane proteins

##### 4.1.2.1. GABA receptors

The most abundant inhibitory neurotransmitter of the central nervous system is γ-aminobutyric acid (GABA) and the GABA-A receptor (GABAR) is the target of the majority of commercially developed general anesthetic agents, with the notable exception of ketamine and nitrous oxide. Activation of the GABA-B receptor can also influence neuronal excitability but is not thought to contribute significantly to the anesthetic action of conventional anesthetics (Brohan and Goudra, 2017). There were no studies identified that reported results for the GABA-B receptor.

In a study of autaptic cultured hippocampal cells, the authors reported that 80% xenon did not alter the peak, total current or inhibitory current dynamics observed following electrical stimulation of inhibitory neurons when compared to control (de Sousa et al., 2000). The authors also reported that the current observed following bath application of GABA 3μM was also unaffected by xenon.

Three studies have investigated the effects of xenon on murine GABAR in transfected cell lines with somewhat conflicting results. All three studies investigated the α1, β2, γ2 subunit composition in either HEK 293 or xenopus oocytes. One study reported that a solution saturated with xenon at room temperature increased ionic current evoked by bath application of GABA (Hapfelmeier et al., 2000). They noted that the potentiation was only present at lower doses of GABA (10^−7^-10^−5^ M) and that no potentiation was seen at higher doses. Another study also reported that 46% xenon significantly potentiated currents measured following bath application of GABA (at a concentration of EC5 of maximal response) (Yamakura and Harris, 2000). In contrast, another study reported that 80% xenon had no effect on the current induced by bath application of 10μM GABA (10^−5^ M) (Gruss et al., 2004).

Four subsequent studies have investigated the effect of xenon on ionic current through GABAR in murine tissue slices (Haseneder et al., 2008, 2009a; Georgiev et al., 2010; Yamamoto et al., 2012). These studies were performed in both brain (prefrontal cortex, basolateral amygdala) and spinal cord slices (dorsal and ventral horn) and xenon concentration was between 50 and 65%. Currents were evoked either by electrical stimulation or application of GABA, in concentrations of 0.5–1 mM. All four studies reported that xenon did not affect peak or total charge transfer through GABAR. Two of these studies also reported the effect of xenon on miniature inhibitory postsynaptic currents (IPSC), in the presence of tetrodotoxin, and reported that 50% xenon had no effect on either the amplitude or frequency of miniature IPSC (Georgiev et al., 2010; Yamamoto et al., 2012).

Two studies have utilized the single bouton preparation, from hippocampal CA3 neurons and neurons of the sacral dorsal commissural nucleus, to investigate the effect of xenon on ionic current through GABAR (Nonaka et al., 2019; Kubota et al., 2020). In both studies, 70% xenon had no effect on the current induced by bath application of 10^−5^ M of GABA. Both studies investigated the effects of 70% xenon on spontaneous IPSC and both reported that xenon reduced the frequency, but not the amplitude of spontaneous IPSC. Both studies also reported that xenon increased the failure rate and paired pulse ratio of currents induced by paired pulse electrical activation, suggesting a presynaptic site of action.

##### 4.1.2.2. Glycine receptors

Glycine receptor activation leads to neuronal hyperpolarization and inhibitory glycine function is critical to many physiological processes including control of muscle tone and sensory processing. The glycine receptor is also modulated by several conventional anesthetics and ethanol (Burgos et al., 2016).

In two cell transfection studies, xenon in concentrations from 40 to 70% were reported to increase ionic current through glycine receptors in response to bath application of glycine when compared to control conditions (Daniels and Roberts, 1998; Yamakura and Harris, 2000). One study reported the effect of a number of volatile anesthetics as well as nitrous oxide. At clinically relevant concentrations they reported that xenon potentiated glycine receptors by 50% whilst volatiles potentiated the response by 200% and nitrous oxide by 75%.

In contrast, two studies of spinal cord slices suggest xenon has negligible effect on glycine receptors. In an *in vitro* spinal cord tissue slice, 50% xenon did not affect the ionic current evoked in lamina IX neurons by bath application of 0.5 mM of glycine (Yamamoto et al., 2012). In an *in vivo* model, the ionic current measured in substantia gelatinosa neurons, activated following bath application of 1 mM of glycine to the lumbosacral spinal cord in an anesthetized rat model, was not affected by the presence or absence of 50% xenon (Georgiev et al., 2010).

##### 4.1.2.3. Other ligand gated membrane proteins

Several general anesthetic agents interact with the nicotinic acetylcholine receptor (nAChR), although to date there is animal data to suggest these interactions are essential to their anesthetic action (Chau, 2010). Activation of nAChR leads to inward flux of positive charge, mainly sodium, and depolarization of the cell.

The ability of xenon to interact with nAChR was investigated in two studies. Both were performed in xenopus oocytes transfected with different receptor subtypes, the heteromeric α4β2 and the homomeric α7 (Yamakura and Harris, 2000; Suzuki et al., 2002). Both studies reported a significant reduction in ionic current on exposure to xenon. The reduction was dose dependent in the study of the homomeric α7 subtype.

One study of xenopus oocytes transfected with human 5-hydroxytryptamine 3A (5HT3A) receptors reported that xenon inhibited ionic current in a competitive, dose dependent manner, with ionic current reduced in response to 5-hydroxytryptamine agonist to around 50% and around 20% of control response, in the presence of 30 and 70% xenon, respectively (Suzuki et al., 2002).

#### 4.1.3. Non-ligand gated membrane proteins

##### 4.1.3.1. Potassium channels

The review identified five studies investigating the interaction of xenon with membrane bound potassium channels. The classes of potassium channels investigated included two pore domain potassium channels (P2K), ATP sensitive potassium channels (KATP) and hyperpolarization-activated cyclic nucleotide gated channels (HCN).

Two studies reported that 80% xenon increased ionic current through the human TREK-1 channel expressed in HEK293 cells when compared to control conditions (Gruss et al., 2004; Harris et al., 2013). One study reported that this potentiation of ionic current was significantly reduced in TREK-1 channels in which the C-terminus was truncated (Gruss et al., 2004). Another study reported that xenon was the only gas to exhibit such an effect among several noble gases investigated (Harris et al., 2013). One study also investigated the effects of xenon on TASK-3, a P2K known to be sensitive to volatile anesthetic agents (Gruss et al., 2004). The authors reported that 80% xenon had no effect on ionic current in continuous recordings, or in response to a voltage ramp.

Two studies investigated the effects of xenon on KATP channels (Bantel et al., 2009, 2010). Both studies, from the same authors, were conducted utilizing HEK293 cells transfected with murine subunits of the KATP receptor. Both studies reported that 80% xenon increased current activation of the Kir6.2-SUR1 channel. In contrast, of the volatile agents investigated, including sevoflurane, isoflurane and halothane, all inhibited KATP current activation (Bantel et al., 2009).

The effects of 50% xenon were also reported in the first study. Xenon at this concentration also increased ionic current but the increase was not statistically significant (Bantel et al., 2009). The differential effects of xenon at cell surface and mitochondrial KATP channels was also clarified in this study by the use of specific inhibitors, and the authors reported that xenon's potentiation was specific for plasmalemmal KATP channels (Bantel et al., 2009).

In the second study, the authors reported that xenon potentiation was unchanged in a Kir6.2 mutant that forms active channels in the absence of the SUR1 subunit, suggesting that xenon's effect is due to interaction with the Kir6.2 subunit (Bantel et al., 2010). The authors also investigated the effect of xenon on Kir1.1, an inwardly rectifying potassium channel, and reported that xenon did not alter ionic current through this channel (Bantel et al., 2010).

One study utilized a murine derived neuronal and glial cell culture to investigate the effect of xenon preconditioning on cell viability following an oxygen and glucose deprivation injury (Bantel et al., 2009). The authors reported that cell viability measured 24 h following the injury was completely reversed in cell cultures exposed to 75% xenon for 2 h prior to injury. The ability of xenon to prevent injury was abolished in the presence of the cell surface KATP inhibitor tolbutamide. The authors reported that the ability of sevoflurane to prevent injury was not significantly different in the presence or absence of tolbutamide, suggesting that KATP is not involved in protection provided by sevoflurane.

One study investigated the effect of xenon on the hyperpolarization-activated cyclic nucleotide gated channel 2 (HCN2) utilizing both HEK293 cells transfected with murine HCN2, and thalamocortical tissue slices from both wild type and HCN2 knock-out mice (Mattusch et al., 2015). HCN2 are voltage sensitive, becoming activated at hyperpolarizing voltages and passing an inward current which favors depolarization (Santoro et al., 2000).

The authors reported that in whole cell patch clamps of thalamocortical neurons, 65% xenon decreased the maximum current amplitude and the voltage required to achieve half maximal activation, i.e., greater hyperpolarization was required to achieve the same ionic current flow. The authors also reported that the effects of xenon were abolished in the presence of high concentrations of cyclic AMP.

The authors also reported the effect of xenon on two important features of post-hyperpolarization HCN2 behavior in thalamocortical neurons, sag amplitude and time to rebound burst. Xenon reduced sag amplitude and increased the time to rebound bursting when compared to control (Mattusch et al., 2015). This effect was also abolished in the presence of a high concentration of cAMP. The effects of xenon on current activation and post-hyperpolarization behavior were duplicated in HEK293 cells transfected with murine HCN2.

Finally, the authors utilized a voltage sensitive dye imaging technique to measure cortical depolarization following activation of thalamocortical neurons. The authors reported that 65% xenon reduced cortical depolarization when compared to control. This effect was not observed in HCN2 knock-out mice, suggesting that HCN2 was responsible for the observed effects of xenon on cortical depolarization (Mattusch et al., 2015).

##### 4.1.3.2. Calcium channels

Given its crucial role in neurotransmitter release, second messenger signaling, and potential as a cellular toxin, the concentration of intracellular calcium is tightly controlled within neurons. Both plasmalemmal calcium ATPase (PMCA) and voltage gated calcium channels (VGCC) have been investigated as potential targets for anesthetic action and neuroprotectants (Yamakage and Namiki, 2002; Kopecky et al., 2014).

The review identified four studies in which the effect of xenon on PMCA was reported. Three of these studies utilized synaptosomes from murine neurons which were exposed to helium or xenon (Franks et al., 1995; Horn et al., 1995; Singh et al., 1995). In these studies, xenon was reported to reduce the activity of PMCA in a dose dependent manner in the range of 0.5–1.5 atm (Singh et al., 1995).

Xenon also increased calcium uptake measured by calcium fluorescence in murine cortical cultures (Franks J. J. et al., 1998). Whilst 20 and 40% xenon in solution had no effect on baseline or evoked calcium uptake, xenon at 60 and 80% significantly reduced calcium uptake at baseline and following NMDA administration.

Xenon at a concentration of 80% had no effect on ionic current flow in response to a depolarizing voltage step protocol when compared to control conditions in HEK293 cells transfected with N-type calcium channels (White et al., 2005). Xenon also had no effect on current transfer in response to depolarizing voltage steps in CA1 pyramidal neurons in the presence of pharmacological isolation of the L-type channel (Kratzer et al., 2012). Finally, 70% xenon had no effect on the barium current (a surrogate for calcium current) in response to depolarization of a sacral dorsal commissural nucleus neuron (Kubota et al., 2020).

##### 4.1.3.3. Monoamine transporters

The monoamines dopamine and norepinephrine are neurochemical modulators that are important regulators of sleep and wakefulness and have been identified as potential mediators of the anesthetic effect of several commonly utilized anesthetic agents (Purdon et al., 2015).

Two studies investigated dopamine transport in a murine brain slice preparations exposed to xenon and control gases in a recording chamber. In the first study, dopamine release in nucleus accumbens was recorded following amphetamine and potassium chloride stimulation (David et al., 2006). Xenon at 50 and 75% reduced dopamine release in response to amphetamine, whilst 75% xenon increased dopamine release in response to potassium chloride and 50% xenon had no effect.

In the second study, striatal brain slices were exposed to an oxygen-glucose deprivation injury either in the presence of various concentrations of xenon gas (balanced with nitrogen) or nitrogen alone (David et al., 2008). Dopamine release was significantly reduced in slices exposed to xenon ranging from 25 to 75%. Release in the presence of 15% xenon was not significantly different from controls. The response of slices to subsequent exposure to potassium chloride was similar for control and xenon treated slices.

One study investigated the effect of xenon on norepinephrine uptake utilizing two transfected cell lines, HEK293 transfected with human norepinephrine transporter (NET) and SH-SY5Y transfected with both human NET and NMDAR (Neukirchen et al., 2012). Utilizing fluorometric measurement, the authors reported that 65% xenon had no effect on xenon uptake in the HEK293 cell lines expressing only NET. In contrast, xenon increased uptake of NE in the SH-SY5Y expressing both NET and NMDAR. The effect was non-significant for 32.5% xenon, whilst 50 and 65% resulted in significantly reduced uptake. This was confirmed utilizing radiometric analysis.

##### 4.1.3.4. Other membrane bound proteins

One study investigated the interaction between xenon and voltage-gated sodium channels in a single bouton preparation of the sacral commissural nucleus of the spinal cord and reported that 70% xenon had no effect on ionic current (Kubota et al., 2020).

Acid sensing ion channels (ASIC) are sodium channels, insensitive to voltage but sensitive to pH changes, that might have a role in regulating neurotransmitter release. In xenopus oocytes transfected with various rat ASIC subtypes, One study reported that xenon reduced the decay time of currents induced by acidic environments for the three ASIC subtypes expressed in the CNS, as well as reducing current amplitude for the ASIC 1α homomeric subtype (Lehmke et al., 2018).

One study investigated the effect of xenon on the transient receptor potential vanilloid 1 (TRPV1) receptor in cultures of murine primary sensory neurons, and in HEK293 cells transfected with human TRPV1. Whilst traditionally associated with the capsaicin evoked response in the peripheral nervous system, TRPV1 is also widely expressed in the CNS (Ho et al., 2012). The authors reported that 75% xenon reduced cobalt uptake, a measure of TRPV1 activation, only at low doses of capsaicin (White et al., 2011).

### 4.2. Intracellular signaling pathways

#### 4.2.1. Neurotrophic factors

The review identified three studies that quantified the expression of brain derived neurotrophic factor (BDNF) (Ma et al., 2006; Peng et al., 2013; Dandekar et al., 2018), a neurotrophic factor that activates a number of the kinases involved in pro-survival signaling pathways through its interaction with the tyrosine kinase B receptor (Bathina and Das, 2015). All three of these studies reported an increase in BDNF expression, although in one study this did not achieve statistical significance (Dandekar et al., 2018).

#### 4.2.2. PI3K/Akt pathway

The PI3K/Akt pathway is an intracellular transduction pathway, activated by tyrosine kinases, which regulates many processes such as cell proliferation, metabolism and has been the target of several neuroprotective agents (Long et al., 2021).

In four of the five studies that quantified phosphorylated Akt (Akt-p) expression, the authors reported an increase following xenon exposure (Limatola et al., 2010; Peng et al., 2013; Liu et al., 2016; Fan et al., 2021).

Two of these studies also quantified non-phosphorylated Akt and had conflicting results. In a stroke model, one study reported a significant increase in brain Akt expression following the injection of xenon containing liposomes (Peng et al., 2013). In another study, the authors reported no change in Akt expression in the spinal cord following an ischemic spinal cord injury followed by 2 h of 50% xenon treatment (Fan et al., 2021).

Two studies reported the effect of xenon on expression of phosphatidylinositol-3 kinase (PI3K) with conflicting results. One study identified an increase in phosphorylated PI3K (PI3K-p) and no change in the non-phosphorylated form (Fan et al., 2021). In another study, the authors found no difference in PI3K brain expression following tibial fracture and fixation under isoflurane anesthesia in a group that received 50% xenon anesthesia for 20 minutes (Vizcaychipi et al., 2011).

#### 4.2.3. Mitogen activated protein kinase pathway

The mitogen activated protein kinase (MAPK) pathway is another key signaling pathway regulating cellular processes and survival. Upstream activation of the pathway occurs in response to a wide array of extra and intracellular signals, including receptor tyrosine kinases and the activation of protein kinase C (Guo et al., 2020).

In two studies in which an ischemic injury model was utilized, one in spinal cord and the other in brain tissue, the quantity of phosphorylated MAPK (MPAK-p) was greater in the xenon treatment group when compared to controls (Peng et al., 2013; Liu et al., 2016). In one of these studies, the expression of the individual phosphorylated MAP kinases, p44 and p42, was reported (Peng et al., 2013).

In a non-injury study and in a study in which xenon was administered 24 h prior to an isoflurane and nitrous oxide exposure, the authors reported no difference in MAPK-p expression when compared to controls (Shu et al., 2010; Dandekar et al., 2018).

#### 4.2.4. Downstream proteins involved in cell survival signaling

The review identified six studies that quantified the expression of B-cell lymphoma protein (Bcl-2), a pro-survival protein, following xenon exposure. The result of all six studies reported that xenon administration was associated with an increase in expression of Bcl-2 (Ma et al., 2006; Shu et al., 2010; Zhuang et al., 2012; Yang et al., 2014; Zhao et al., 2018; Jin et al., 2021).

Three of these studies also reported the effect of xenon on the expression of the pro-apoptotic Bcl-2-associated X protein (Bax) (Zhuang et al., 2012; Yang et al., 2014; Jin et al., 2021). In all three studies, all of which included an injury model in brain or spinal cord, xenon exposure was associated with reduced expression of Bax.

In one study, a 30-min exposure of non-injured rats to 55% xenon resulted in increased expression of GSK3β, a Bax inhibitor, in brain tissues (Kuzovlev et al., 2021).

Three studies reported the effect of xenon treatment on expression of cytochrome c (Cyt-c), a protein released from mitochondria as part of the mitochondrial apoptosis pathway. One study of a spinal cord ischemia and reperfusion model reported that 1-h of 50% inhaled xenon after injury reduced the expression of Cyt-c in the spinal cord (Yang et al., 2014). The other two studies, a non-injury study and an anesthetic exposure study (xenon administered 24 h prior to isoflurane and nitrous oxide exposure), showed no change in Cyt-c expression in brain tissues (Ma et al., 2007; Shu et al., 2010).

All three studies also reported the expression of caspase 3. Both the spinal cord ischemia and anesthetic injury studies reported that xenon exposure reduced the expression of caspase 3 (Shu et al., 2010; Yang et al., 2014), whilst the non-injury study identified no change (Ma et al., 2007). The non-injury study, in which rats were sacrificed immediately following inhalation of 75% xenon for 6 h, also reported no significant change in expression of caspases 8 and 9 following xenon exposure.

The abundance of phosphorylated cAMP response element binding protein (CREB-p) in either brain or spinal cord was investigated in three studies (Ma et al., 2006; Luo et al., 2008; Fan et al., 2021). These studies were performed in both injured and non-injured rats. Xenon treatment was delivered pre-injury in one study (Ma et al., 2006). In all three studies, xenon exposure was associated with increased expression of CREB-p.

The review also identified one study that reported an increase in hypoxia induced factor 1α, when xenon was given prior to an ischemic cerebral injury (Limatola et al., 2010) and another that reported increased cerebral expression of activity dependent neuroprotective protein (ADNP) following xenon exposure in non-injured rats (Cattano et al., 2008).

One study identified that expression of the p53 oncogene was reduced in rats pretreated with 70% xenon inhalation 24 h prior to anesthetic gas exposure (Shu et al., 2010). Another study identified a reduction in the phosphorylated form of NMDAR in the ipsilateral dorsal horn of rats when exposed to xenon inhalation following formalin injection of the hind paw, a model of pain-induced central sensitization (Fukuda et al., 2002).

#### 4.2.5. Calcium signaling and protein synthesis

In two studies, the intracellular calcium chelator BAPTA was reported to prevent xenon rescue of cultured cells in hypoxic conditions (Petzelt et al., 2003, 2004). BAPTA alone, in non-hypoxic controls, did not induce cellular injury. In contrast, KN-93, an inhibitor of the Ca^2+^/calmodulin-dependent protein kinase II (CaMK-II) complex did not interfere with the ability of xenon to rescue hypoxic cortical neurons (Petzelt et al., 2003). KN-93 alone provided a neuroprotective effect in hypoxic neurons in this study, although significantly less than that provided by xenon.

The review identified one study in which a non-specific inhibitor of protein synthesis was utilized to investigate the relationship between xenon preconditioning and protein synthesis (Ma et al., 2006). The authors reported that xenon preconditioning, 24 h prior to oxygen-glucose deprivation, reduced LDH release in rat cortical cells. This effect was significantly reduced if cycloheximide, a protein synthesis inhibitor, was given during and after xenon administration. The authors reported that their findings suggest that xenon preconditioning depends, at least in part, on the synthesis of neuroprotective proteins.

### 4.3. Gene transcription

The review identified two studies that utilized suppression subtractive hybridization (SSH) to investigate the effect of xenon exposure on gene expression. Both studies describe the results of one experiment in which rats were exposed to 2 h of 75% xenon inhalation. SSH was performed to identify differential gene expression between the xenon group and a control group that inhaled air (Cattano et al., 2008; Valleggi et al., 2008).

The authors reported that 49 genes were differentially expressed in the xenon exposure group. The authors then selected 11 of these genes, based on their “predicted biologic properties” to quantify by reverse transcription PCR (RT PCR). The authors reported that six of these genes were significantly upregulated, including activity dependent neuroprotective protein (ADNP) and other genes for proteins involved in apoptosis and NMDA trafficking (Cattano et al., 2008; Valleggi et al., 2008). In another study derived from the same experiment, the authors reported that mRNA expression was reduced for Akt and increased for c-Jun N-terminal kinase kinase 1 (JNKK1), a downstream mediator of the MAPK pathway, quantified by RT PCR (Cattano et al., 2011).

Two studies utilized RT PCR to quantify mRNA expression following xenon exposure in an injury model. The authors reported that the expression of the mRNA for the proteolytic enzyme membrane metalloproteinase 9 (MMP9) was significantly reduced in the xenon group (Metaxa et al., 2014). As well as its ability to degrade cells, MMP9 is also reported to have a role in NMDAR trafficking (Michaluk et al., 2009).

One study utilized RT PCR to quantify the expression of mRNA for chloride intracellular channel 4 (CLIC4). This widely expressed intracellular channel can induce apoptosis when overexpressed. The authors reported that 3 h inhalation of 50% xenon significantly reduced expression of CLIC-4 in a model of white matter damage induced by lipopolysaccharide injection in rats (Yin et al., 2018).

The review identified one study that reported the results of a multiplex gene expression method that utilizes fluorescent barcoding of target genes to quantify mRNA expression (Filev et al., 2021). The authors identified 91 target genes of interest and reported multiplex gene expression in rats following traumatic brain injury. The authors reported that in the contralateral (to injury) samples, xenon treatment induced the expression of the inflammatory protein interferon regulatory factor 1 (Irf1) and the antioxidant heme oxygenase 1 (Hmox1). The importance of the two other proteins identified as upregulated, Myd88 and Tlr2 is unclear. After correction for multiple comparisons, no statistically significant changes were reported in the damaged brain area.

## 5. Discussion

**Figure 2:**
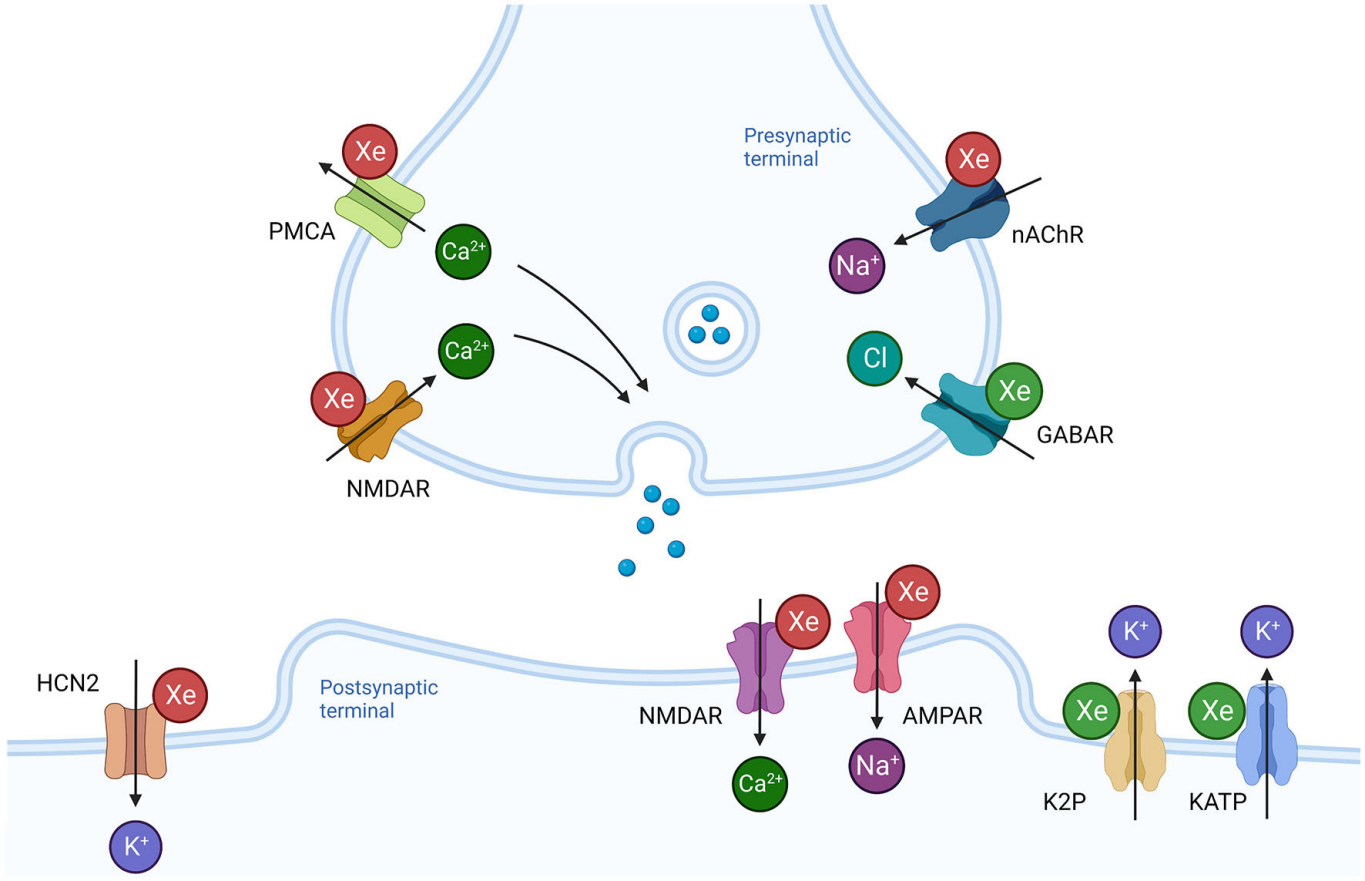
Effects of xenon on synaptic transmission and neuronal excitability. Xenon influences a number of membrane bound proteins present in the presynaptic terminal that regulate neurotransmitter release. Inhibition of the nicotinic acetylcholine receptor (nAChR) would prevent depolarization and reduce the probability of neurotransmitter release. Inhibition of presynaptic NMDAR and potentiation of GABAR would also reduce probability of neurotransmitter release. In contrast, inhibition of plasmalemmal calcium ATPase (PMCA) would promote release. Xenon reduces ionic current through NMDAR and AMPAR at the postsynaptic terminal. Potentiation of hyperpolarizing ionic currents through potassium channels (K2P, KATP) and inhibition of the depolarizing ionic current through the hyperpolarization activated cyclic nucleotide channel 2 (HCN2) reduce neuronal membrane excitability. Green Xe = xenon increases ionic current, Red Xe = xenon reduces ionic current. Created with BioRender.com.

**Figure 3:**
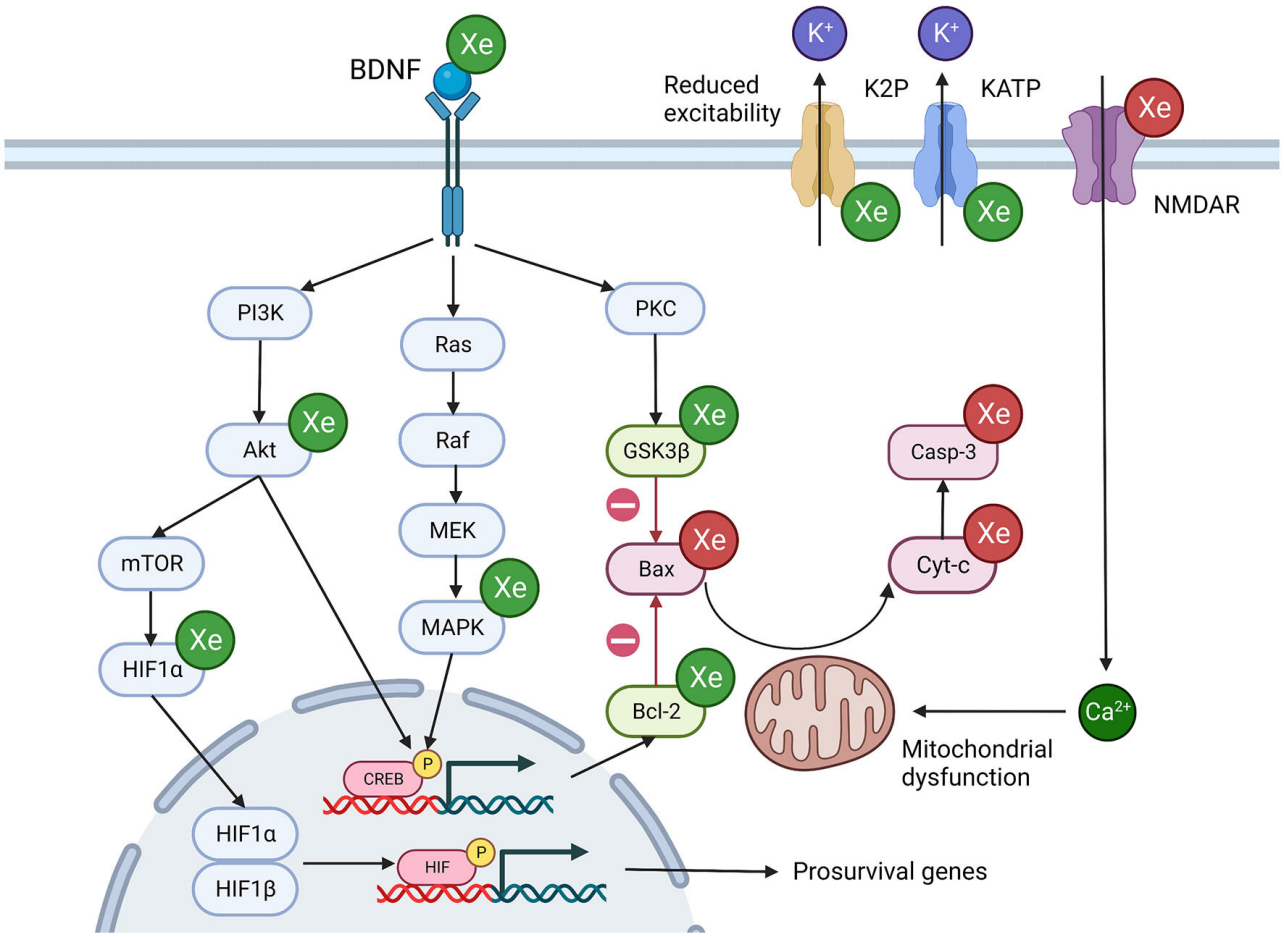
Potential mechanisms of xenon induced neuroprotection. Xenon influences multiple overlapping cellular processes that promote cell survival. Green Xe = xenon increases protein expression/ionic current, Red Xe = xenon reduces protein expression/ionic current, Green protein = pro-survival protein, Red protein = pro-apoptosis protein. Akt, Ak strain transforming protein; Bcl-2, B-cell lymphoma protein; Bax, Bcl-2-associated X protein; BDNF, Brain derived neurotrophic factor; CREB, cAMP response element binding protein; Cyt-c, Cytochrome c; GSK3β, Glycogen synthase kinase 3 beta; Hmox1, Heme oxygenase 1; MAPK, Mitogen activated protein kinase; mTOR, Mammalian target of rapamycin; NMDA, N-methyl-D-aspartatel; PKC, Protein Kinase C. Created with BioRender.com.

The review identified multiple cellular targets of xenon including membrane bound proteins, intracellular signaling pathways and gene transcription factors. The relevance of these interactions to anesthesia and neuroprotection are discussed below. A graphical representation of the cellular targets responsible for the anesthetic and neuroprotective actions of xenon are also presented in Figures 2, 3.

### 5.1. Mechanisms of xenon anesthesia

#### 5.1.1. Ionotropic glutamate receptors

The first report pertaining to the action of xenon at ligand gated receptors identified that xenon reduced ionic current through NMDAR (Franks N. P. et al., 1998). Subsequent investigations have confirmed that xenon antagonizes the NMDAR at multiple brain and spinal cord sites in the mammalian CNS (Table 3). Xenon antagonism has also been reported in all four NR2 subtypes of the NMDAR.

There is good evidence that xenon acts as a non-competitive inhibitor of all three ionotropic glutamate receptor subtypes: NMDAR, AMPAR and KAR. This has led some authors to suggest that all three receptors might share an allosteric binding site for xenon (Nonaka et al., 2019; Kubota et al., 2020). This is supported by structural studies that suggest that the ligand binding domain is highly conserved between the three subtypes (Liu et al., 2010).

Xenon also has an additional site of action at the glycine binding site of the NMDAR. Xenon competes with the NMDAR co-agonist glycine at this site and antagonizes channel opening. This competitive inhibition of the NMDAR distinguishes xenon from nitrous oxide (Mennerick et al., 1998) and ketamine (Zhang et al., 2021). The distinct nature of xenon binding to the NMDAR is reflected in its electrophysiological and behavioral effects.

The binding pocket for ketamine lies within the central vestibule of the NMDAR and binding requires that the channel is in the open configuration (Zhang et al., 2021). In the cerebral cortex, ketamine preferentially blocks the rapidly opening NMDAR of inhibitory interneurons with subsequent disinhibition of excitatory neurons (Purdon et al., 2015). This increased excitability is reflected in increased power in the beta and gamma frequency bands in the electroencephalogram (EEG) following ketamine administration (Akeju et al., 2016). In contrast, xenon administration is associated with an increase in power in the lower frequency delta and theta band of the EEG (Laitio et al., 2008; McGuigan et al., 2021). This would suggest that the increased excitability of the cortex is a feature of open channel NMDAR blockade and not a mechanism by which xenon induces its anesthetic effect.

Transient slow-delta oscillations have also been reported following the administration of ketamine and nitrous oxide. These have been attributed to a loss of excitatory input from the thalamus and basal forebrain to the cortex (Akeju et al., 2016; Pavone et al., 2016). Whilst this mechanism might be shared by xenon, the persistence of slower oscillations throughout the xenon anesthetic episode (McGuigan et al., 2021) suggest that xenon has distinct mechanisms of action.

Open channel NMDAR blockade, particularly if combined with a slower offset, is also associated with psychomimetic effects (Chen and Lipton, 2006). Psychomimetic effects have not been reported for xenon as they have for open channel blockers such as ketamine, MK-801 and memantine. The nature of xenon antagonism of NMDAR, competition with the co-agonist glycine, appears to be associated with less psychomimetic effects.

#### 5.1.2. Non-ligand gated channels within the thalamus

The hyperpolarization-activated cyclic nucleotide gated channel 2 (HCN2) is one of four isoforms expressed in the CNS. HCN2 is widely expressed in the brain, including the thalamus (Santoro et al., 2000), and has been proposed as a site of action for volatile anesthetics and propofol (Ying et al., 2006; Goldstein, 2015). Although only investigated in one study, multiple methodological approaches identified that xenon inhibited HCN2 activity in both transfected cells and tissue slices (Table 5). The ability of xenon to inhibit HCN2 contrasts with ketamine, which inhibits HCN1 in the cortex and has no effect on the HCN2 isoform (Chen et al., 2009).

HCN2 passes an inward depolarizing current (I_h_ current) in response to hyperpolarization and is thought to be responsible, along with T-type calcium channels, for the burst firing of thalamocortical neurons associated with sleep spindles and the alpha oscillations observed during GABAergic anesthesia (He et al., 2014). The effects of xenon on I_h_ are similar to those reported for propofol (Mattusch et al., 2015). Xenon increased the sag amplitude and delay time to bursting in thalamocortical cells, which has been reported to reduce the oscillation frequency of thalamocortical cells (Ying et al., 2006). This is supported by computational neural models which suggest that a reduction in I_h_ conductance can initiate low frequency oscillations in the thalamus (Soplata et al., 2017).

Xenon is reported to antagonize the NR2C subtype of NMDAR (Weigt et al., 2008) (Table 3) which differs from the more abundant NR2A and NR2B subtypes in both function and distribution (Liu et al., 2019). The neurons of the reticular thalamus preferentially express the NR2C subtype (Liu et al., 2019) and application of the competitive NMDAR antagonist AP5 induces burst firing in reticular thalamus neurons (Zhang et al., 2009). A knockout study confirmed that this action is mediated by NR2C (Zhang et al., 2012). The reticular thalamus provides inhibitory modulation of thalamocortical cells and regulates the thalamocortical oscillations that are associated with altered states of arousal (Clemente-Perez et al., 2017).

The electrophysiological features of xenon are distinct from other NMDAR antagonists. Xenon administration is associated with the dominance of lower frequency delta and theta oscillations which are present throughout the anesthetic episode (Laitio et al., 2008; McGuigan et al., 2021). Given the presence of membrane bound targets for xenon within the thalamus, and the ability of thalamocortical oscillations to disrupt consciousness (Lewis et al., 2015), the anesthetic effect of xenon is likely modulated, at least in part, via the thalamus.

#### 5.1.3. Other potential membrane bound targets

Tissue slice studies of xenon suggest that it has neglible influence on ionic flow through postsynaptic receptors for inhibitory neurotransmitters, such as GABAR and glycine receptors (Table 4). In contrast, two studies of single bouton neuron preparations, designed to investigate pre- and postsynaptic mechanisms, identified a reduction in presynpatic inhibitory neurotransmitter release during xenon exposure. There is also evidence from transfected cell studies that xenon can potentiate GABAR opening at low doses of agonist (Table 4). Taken together, xenon might reduce inhibitory neurotransmission by potentiating presynpatic GABAR, thereby reducing neurotransmitter release. However, given the absence of effect on pharmacologically and electrically evoked inihbitory postsynpatic currents, xenon's effect on presynpatic inhibitory neurotransmitter release is unlikely to contribute significantly to xenon anesthesia.

**Table 4:** Summary of membrane bound proteins—other ligand gated membrane protein results.

**References**	**Model**	**Cell line/tissue**	**Species (receptor)**	**Receptor subtype**	**Xenon dose**	**Outcome reported**	**Direction of effect**
**GABA**							
Hapfelmeier et al. (2000)	Cultured cells	HEK 293	Rat	α1β2/α1β2γ2λ	100%	Ionic current	**↑**↔^*^
Yamakura and Harris (2000)	Cultured cells	Xenopus oocytes	Human	α1β2g2s	46%	Ionic current	**↑**
de Sousa et al. (2000)	Cultured cells	Hippocampal neurones	Rats		80%	Charge transfer	**↔**
Gruss et al. (2004)	Cultured cells	HEK293	Human	α1β2g2s	80%	Ionic current	**↔**
Haseneder et al. (2008)	Tissue slice	Amygdala	Mouse		65%	Ionic current	**↔**
Haseneder et al. (2009b)	Tissue slice	Prefrontal cortex	Mouse		65%	Ionic current	**↔**
Haseneder et al. (2009b)	Tissue slice	Dorsal spinal cord	Mouse		65%	Ionic current	**↔**
Georgiev et al. (2010)	Tissue slice	Dorsal spinal cord	Rat		50%	Ionic current	**↔**
Georgiev et al. (2010)	Tissue slice	Dorsal spinal cord	Rat		50%	Presynaptic transmission	**↔**
Yamamoto et al. (2012)	Tissue slice	Ventral spinal cord	Rat		50%	Ionic current	**↔**
Yamamoto et al. (2012)	Tissue slice	Ventral spinal cord	Rat		50%	Presynaptic transmission	**↔**
Yamamoto et al. (2012)	Tissue slice	Ventral spinal cord	Rat		50%	Postsynaptic transmission	**↔**
Nonaka et al. (2019)	Single bouton	Hippocampal CA3 neurone	Rats		70%	Ionic current	**↔**
Nonaka et al. (2019)	Single bouton	Hippocampal CA3 neurone	Rats		70%	Presynaptic transmission	**↓**
Nonaka et al. (2019)	Single bouton	Hippocampal CA3 neurone	Rats		70%	Postsynaptic transmission	**↔**
Nonaka et al. (2019)	Single bouton	Hippocampal CA3 neurone	Rats		30%	Presynaptic transmission	**↓**
Nonaka et al. (2019)	Single bouton	Hippocampal CA3 neurone	Rats		30%	Postsynaptic transmission	**↔**
Kubota et al. (2020)	Single bouton	SDCN	Rat		70%	Ionic current	**↔**
Kubota et al. (2020)	Single bouton	SDCN	Rat		70%	Presynaptic transmission	**↓**
Kubota et al. (2020)	Single bouton	SDCN	Rat		70%	Postsynaptic transmission	**↔**
**Glycine**							
Daniels and Roberts (1998)	Cultured cells	Xenopus oocytes	Human	α1	40%	Ionic current	**↑**
Daniels and Roberts (1998)	Cultured cells	Xenopus oocytes	Human	α1	70%	Ionic current	**↑**
Yamakura and Harris (2000)	Cultured cells	Xenopus oocytes	Human	α1	46%	Ionic current	**↑**
Georgiev et al. (2010)	Tissue slice	Dorsal spinal cord	Rat		50%	Ionic current	**↔**
Yamamoto et al. (2012)	Tissue slice	Ventral spinal cord	Rat		50%	Ionic current	**↔**
**Other**							
Yamakura and Harris (2000)	Cultured cells	Xenopus oocytes	Rat	nACh α4β2/α4β4	46%	Ionic current	**↓**
Suzuki et al. (2003)	Cultured cells	xenopus oocytes	Human	nACh α7 receptor	35%	Ionic current	**↓**
Suzuki et al. (2002)	Cultured cells	Xenopus oocytes	Human	5HT3α	35-100%	Ionic current	**↓**

Direction of effect: ↑–Increased, ↓–Reduced, ↔–No change.

Color of arrow represents statistics presented by authors: Green—*p*-value in comparison to control given, Amber—no *p*-value but standard error of mean presented for xenon and control groups, Red—*p*-value/SEM not given for xenon and control groups.

Presynaptic transmission include measures of miniature, spontaneous and evoked potentials that indicate presynaptic mechanisms. Postsynaptic transmission include miniature, spontaneous and evoked potentials that indicate postsynaptic mechanisms. Details given within text.

SDCN, Sacral dorsal commissural nucleus.

* Dependent on experimental conditions, see text for details.

Other potential presynaptic targets for xenon are the nicotinic acetylcholine receptor (AChR) and the plasmalemmal calcium ATPase (PMCA). Depolarization at the presynaptic membrane due to nACh activation is reported to be sufficient to influence both excitatory and inhibitory presynaptic neurotransmitter release (McKay et al., 2007). Whilst multiple anesthetic agents are reported to inhibit nACh *in vitro*, there is no animal data to suggest these interactions are essential to their anesthetic action (Chau, 2010). In contrast, the signifcance of PMCA to anesthetic action is supported by resistance to general anesthetic agents in PMCA knockout animal studies (Franks J. J. et al., 1998).

Xenon was reported to inhbit PMCA in isolated synpatic membranes in hyperbaric conditions and in a microfluometry study (Table 5). In contrast, xenon had no effect on voltage-gated calcium channels (VGCC) of various subtypes. Reduced smooth muscle tone and a reduction in cardiac inotropy and chronotropy have been attributed to the inhibtion of VGCC by volatile anesthestics (Campagna et al., 2003). The lack of activity exhbited by xenon at VGCC might explain the greater hemodynamic stability observed during xenon anesthesia (Law et al., 2016).

**Table 5:** Summary of membrane bound proteins—non-ligand gated proteins.

**References**	**Model**	**Cell line/tissue**	**Species (receptor)**	**Receptor subtype**	**Xenon dose**	**Outcome reported**	**Direction of effect**
**Calcium channels**							
Horn et al. (1995)	Cultured cells	Cerebral synaptosomes	Rat	PMCA	124%	Pumping actvity	**↓**
Singh et al. (1995)	Cultured cells	C6 glioma synaptosomes	Rat	PMCA	50–150%	Pumping actvity	**↓**
Franks et al. (1995)	Cultured cells	Cerebral synaptosomes	Rat	PMCA	124%	Pumping actvity	**↓**
Franks J. J. et al. (1998)	Cultured cells	Cerebral cortex	Mouse	PMCA	20/40%	Intracellular calcium	**↔**
Franks J. J. et al. (1998)	Cultured cells	Cerebral cortex	Mouse	PMCA	60/80%	Intracellular calcium	**↑**
White et al. (2005)	Cultured cells	HEK293	Rat	N-type	80%	Ionic current	**↔**
Kratzer et al. (2012)	Tissue slice	CA1 hippocampal neurones	Mouse	L-type	65%	Ionic current	**↔**
Kubota et al. (2020)	Single bouton	SDCN	Rat	Voltage gated	70%	Ionic current	**↔**
**Potassium channels**							
Gruss et al. (2004)	Cultured cells	HEK293	Human	TASK-3	80%	Ionic current	**↔**
Gruss et al. (2004)	Cultured cells	HEK293	Human	TREK-1	80%	Ionic current	**↑**
Bantel et al. (2009)	Cultured cells	HEK293	Mouse/Rat	KATP (Kir6.2/SUR1)	50%	Ionic current	**↔**
Bantel et al. (2009)	Cultured cells	HEK293	Mouse/Rat	KATP (Kir6.2/SUR1)	80%	Ionic current	**↑**
Bantel et al. (2010)	Cultured cells	HEK293	Mouse/Rat	KATP (Kir6.2/SUR1)	80%	Ionic current	**↑**
Bantel et al. (2010)	Cultured cells	HEK293	Mouse/Rat	Kir1.1	80%	Ionic current	**↔**
Harris et al. (2013)	Cultured cells	HEK293	Human	TREK-1	80%	Ionic current	**↑**
Mattusch et al. (2015)	Cultured cells	HEK293	Mouse	HCN2	65%	Ionic current	**↓**
Mattusch et al. (2015)	Tissue slice	Cortex	Mouse	HCN2	65%	Ionic current	**↓**
Mattusch et al. (2015)	Tissue slice	Cortex	Mouse	HCN2	65%	Signal propagation	**↓**
**Monoamine transport**							
David et al. (2006)	Tissue slice	Nucleus accumbens	Rat	DAT	50–75%	DA release	**↓**
David et al. (2008)	Tissue slice	Corpus striatum	Rat	DAT	25–75%	DA release	**↓**
Neukirchen et al. (2012)	Cultured cells	HEK293	Human	NET	65%	NA uptake	**↔**
Neukirchen et al. (2012)	Cultured cells	SH-SY5Y	Human	NMDA/NET	50%/65%	NA uptake	**↓**
**Others**							
White et al. (2011)	Cultured cells	Primary sensory neurones	Rat	TRPV1	75%	Cobalt uptake	**↓**
White et al. (2011)	Cultured cells	HEK293	Human	TRPV1	75%	Cobalt uptake	**↓**
Lehmke et al. (2018)	Cultured cells	Xenopus oocytes	Rat	ASIC	71%	Ionic current	**↓**
Kubota et al. (2020)	Single bouton	SDCN	Rat	Voltage gated Na^+^	70%	Ionic current	**↔**

Direction of effect: ↑–Increased, ↓–Reduced, ↔–No change.

Color of arrow represents statistics presented by authors: Green—*p*-value in comparison to control given, Amber—no *p*-value but standard error of mean presented for xenon and control groups, Red—*p*-value/SEM not given for xenon and control groups.

SDCN, Sacral dorsal commissural nucleus.

The relevance of two pore postassium channels to the general anesthetic action of volatile agents is supported by a reduction in anesthetic sensitivity in knockout mice (Mathie et al., 2021). Xenon potentiates TREK-1 ionic currents at a magnitude similar to that reported for halothane (Table 5). The ability of xenon to potentiate TREK-1 distinguishes it from other noble gases such as argon, krypton, neon and helium (Harris et al., 2013). Given the absence of TREK-1 activity in other noble gases, TREK-1 potentiation might contribute to xenon's greater potency.

### 5.2. Mechanisms of xenon neuroprotection

#### 5.2.1. Reduced excitation via membrane bound proteins

The NMDAR is the only ionotropic glutamate subtype that is permeable to the bivalent cation calcium. The calcium influx associated with NMDA activation is important to synaptic transmission and is also implicated in many physiological and pathological intracellular processes, including the excitotoxicity associated with both acute and chronic neural injury (Li and Wang, 2016).

The relevance of NMDAR antagonism to xenon neuroprotection was emphasized by three studies which identified that xenon neuroprotection against acute cellular injury was abolished by the co-agonist glycine (**Table 8**). Interestingly, glycine did not abolish xenon protection against a model of low-level excitotoxicity. This suggests that xenon neuroprotection occurs via multiple pathways, some of which are independent of its action at NMDAR.

Dopamine is also thought to play a role in NMDAinduced neurotoxicity (Ma et al., 2002). A reduction in dopamine release in brain slices, in both oxygen-glucose deprived and non-injured brain tissue slices, was attributed to the action of xenon in Table 5. These studies were performed in conditions that would promote dopamine release via the dopamine transporter (DAT). Although it is not known if xenon reduced release of dopamine, or enhanced reuptake via the DAT, limiting excessive dopamine release in the extra-neuronal space might contribute to xenon neuroprotection (Leviel, 2011; Ares-Santos et al., 2013).

As discussed above, xenon potentiates the TREK-1 two pore potassium channel to a similar degree as volatile anesthetic agents (Table 5). The neuroprotective effects of sevoflurane have been attributed to TREK-1 potentiation in a knockout animal study (Tong et al., 2013). The ability to potentiate TREK-1 distinguishes xenon from other noble gases which are less potent neuroprotectants (Harris et al., 2013). It would appear that TREK-1 activity is a neuroprotective mechanism shared by xenon and volatile agents, but not other noble gases.

Xenon's ability to potentiate the potassium ATPase (KATP) channel distinguishes it from volatile agents, which tend to inhibit the channel (Table 5). The function of the KATP, which links membrane potential to the internal metabolic state of the cell, provides an intuitive basis for a role in neuroprotection. This is supported by an injury quantification study in which selective blockade of the receptor abolished xenon's protective effect (**Table 8**). Whilst these findings are based on a limited number of studies, potentiation of the KATP channel would appear to be a mechanism unique to xenon.

#### 5.2.2. Interaction with intracellular protein signaling cascades

The properties that enable xenon to interact with cell membrane bound proteins are equally applicable to intracellular globular proteins (Colloc'h et al., 2007), and xenon altered the activity of multiple intracellular protein cascades.

There is strong evidence that xenon exposure alters the expression of the B-cell lymphocyte 2 (Bcl-2) family of proteins (Table 6). These intracellular proteins mediate the mitochondrial pathway of apoptosis, the primary mode of apoptosis in vertebrates (Wang and Youle, 2009). Activation of the pro-apoptotic Bax protein results in the formation of mitochondrial pores which allow the translocation of cytochrome c into the cytosol, setting off a proteolytic cascade. The pro-survival protein Bcl-2 is one of the major pro-survival proteins which prevents Bax activation (Westphal et al., 2014).

**Table 6:** Protein quantification summary.

**References**	**Protein**	**Tissue**	**Species**	**Injury model**	**Route xenon**	**Xenon dose**	**Xenon duration**	**Direction of effect**
Cattano et al. (2008)	ADNP	Brain	Rat	No	Inhaled	75%	2 h	**↑**
Peng et al. (2013)	Akt	Brain	Rat	Yes	Injected	7 mg.kg^−1^	N/A	**↑**
Dandekar et al. (2018)	Akt	Brain	Rat	No	Injected	6/9 mg.kg^−1^	N/A	**↔**
Fan et al. (2021)	Akt	Spinal cord	Rat	Yes	Inhaled	50%	2 h	**↔**
Limatola et al. (2010)	Akt-p	Brain	Mouse	Yes	Inhaled	70%	2 h	**↑**
Peng et al. (2013)	Akt-p	Brain	Rat	Yes	Injected	7 mg.kg^−1^	N/A	**↔**
Liu et al. (2016)	Akt-p	Spinal cord	Rat	Yes	Inhaled	50%	1 h	**↑**
Dandekar et al. (2018)	Akt-p	Brain	Rat	No	Injected	6/9 mg.kg^−1^	N/A	**↔**
Fan et al. (2021)	Akt-p	Spinal cord	Rat	Yes	Inhaled	50%	2 h	**↑**
Zhuang et al. (2012)	Bax	Brain	Rat	Yes	Inhaled	70%	1.5 h	**↓**
Yang et al. (2014)	Bax	Spinal cord	Rat	Yes	Inhaled	50%	1 h	**↓**
Jin et al. (2021)	Bax	Brain	Mouse	Yes	Injected	200 μL	N/A	**↓**
Ma et al. (2006)	Bcl-2	Brain	Rat	No	Inhaled	70%	2 h	**↑**
Shu et al. (2010)	Bcl-2	Brain	Rat	Yes	Inhaled	70%	2 h	**↑**
Zhuang et al. (2012)	Bcl-2	Brain	Rat	Yes	Inhaled	70%	1.5 h	**↑**
Yang et al. (2014)	Bcl-2	Spinal cord	Rat	Yes	Inhaled	50%	1 h	**↑**
Zhao et al. (2018)	Bcl-2	Brain	Rat	Yes	Inhaled	50%	3 h	**↑**
Jin et al. (2021)	Bcl-2	Brain	Mouse	Yes	Injected	200 μL	N/A	**↑**
Ma et al. (2006)	BDNF	Brain	Rat	No	Inhaled	70%	2 h	**↑**
Peng et al. (2013)	BDNF	Brain	Rat	Yes	Injected	7 mg.kg^−1^	N/A	**↑**
Dandekar et al. (2018)	BDNF	Brain	Rat	No	Injected	6/9 mg.kg^−1^	N/A	**↔**
Ma et al. (2007)	Caspase 3	Brain	Rat	No	Inhaled	75%	6 h	**↔**
Shu et al. (2010)	Caspase 3	Brain	Rat	Yes	Inhaled	70%	2 h	**↓**
Yang et al. (2014)	Caspase 3	Spinal cord	Rat	Yes	Inhaled	50%	1 h	**↓**
Jin et al. (2021)	Caspase 3	Brain	Mouse	Yes	Injected	200 μL	N/A	**↓**
Ma et al. (2007)	Caspase 8	Brain	Rat	No	Inhaled	75%	6 h	**↔**
Ma et al. (2007)	Caspase 9	Brain	Rat	No	Inhaled	75%	6 h	**↔**
Fan et al. (2021)	CREB	Spinal cord	Rat	Yes	Inhaled	50%	2 h	**↔**
Ma et al. (2006)	CREB-p	Brain	Rat	No	Inhaled	70%	2 h	**↑**
Luo et al. (2008)	CREB-p	Brain	Rat	Yes	Inhaled	75%	2 h	**↑**
Fan et al. (2021)	CREB-p	Spinal cord	Rat	Yes	Inhaled	50%	2 h	**↑**
Ma et al. (2007)	Cyt-c	Brain	Rat	No	Inhaled	75%	6 h	**↔**
Shu et al. (2010)	Cyt-c	Brain	Rat	Yes	Inhaled	70%	2 h	**↔**
Yang et al. (2014)	Cyt-c	Spinal cord	Rat	Yes	Inhaled	50%	1 h	**↓**
Kuzovlev et al. (2021)	GSK3β	Brain	Rat	No	Inhaled	55%	30 min	**↔**
Kuzovlev et al. (2021)	GSK3β-p	Brain	Rat	No	Inhaled	55%	30 min	**↑**
Limatola et al. (2010)	HIF1a	Brain	Mouse	Yes	Inhaled	70%	2 h	**↑**
Vizcaychipi et al. (2011)	Hsp72	Brain	Mouse	Yes	Inhaled	70%	20 min	**↔**
Dandekar et al. (2018)	MAPK	Brain	Rat	No	Injected	6/9 mg.kg^−1^	N/A	**↔**
Dandekar et al. (2018)	MAPK-p	Brain	Rat	No	Injected	6/9 mg.kg^−1^	N/A	**↔**
Liu et al. (2016)	MAPK-p	Spinal cord	Rat	Yes	Inhaled	50%	1 h	**↑**
Peng et al. (2013)	MAPK-p	Brain	Rat	Yes	Injected	7 mg.kg^−1^	N/A	**↑**
Shu et al. (2010)	MAPK-p	Brain	Rat	Yes	Inhaled	70%	2 h	**↔**
Peng et al. (2013)	MAPK-p42	Brain	Rat	Yes	Injected	7 mg.kg^−1^	N/A	**↑**
Peng et al. (2013)	MAPK-p44	Brain	Rat	Yes	Injected	7 mg.kg^−1^	N/A	**↔**
Dandekar et al. (2018)	mTOR	Brain	Rat	No	Injected	6/9 mg.kg^−1^	N/A	**↔**
Dandekar et al. (2018)	mTOR-p	Brain	Rat	No	Injected	6/9 mg.kg^−1^	N/A	**↔**
Fukuda et al. (2002)	NMDA-p	Brain	Rat	Yes	Inhaled	79%	20 min	**↓**
Shu et al. (2010)	p53	Brain	Rat	Yes	Inhaled	70%	2 h	**↓**
Vizcaychipi et al. (2011)	PI3K	Brain	Mouse	Yes	Inhaled	70%	20 min	**↔**
Fan et al. (2021)	PI3K	Spinal cord	Rat	Yes	Inhaled	50%	2 h	**↔**
Fan et al. (2021)	PI3K-p	Spinal cord	Rat	Yes	Inhaled	50%	2 h	**↑**
Dandekar et al. (2018)	PKC	Brain	Rat	No	Injected	6/9 mg.kg^−1^	N/A	**↔**
Dandekar et al. (2018)	PKC-p	Brain	Rat	No	Injected	6/9 mg.kg^−1^	N/A	**↔**

Direction of effect (achieved statistical significance): ↑–Increased, ↓–Reduced, ↔–No change. Increases highlighted green. Reductions highlighted red.

Results quantifying the expression of downstream proteins such as cytochrome c (Cyt-c) and caspases of the mitochondrial apoptosis cascade were less consistent and appear to be influenced by the presence or absence of an injury stimulus (Table 6). Interestingly, increases in Cyt-c and caspases 8 and 9 were identified in animals following exposure to clinically relevant concentrations of isoflurane and nitrous oxide (Ma et al., 2007). In contrast, exposure to xenon did not activate these downstream mediators of the mitochondrial pathway of apoptosis.

The PI3K/Akt pathway is an upstream mediator of Bcl-2 family activity. Phosphorylation of Akt promotes phosphorylation of Bcl-2 family proteins, thereby promoting cell survival (Liu et al., 2016; Miao et al., 2016). There is good evidence that inhalational exposure to xenon, pre-injury or post-injury, increases the expression of phosphorylated Akt in animal models (Table 6). One study also identified increased expression of PI3K-p in the xenon treatment group following a spinal cord injury (Fan et al., 2021). The importance of the PI3K/Akt pathway is supported by evidence that a PI3K/Akt pathway inhibitor could abolish the protective effect of xenon (Liu et al., 2016).

There is evidence that when given after a hypoxic-ischemic injury, xenon can influence the activity of another major protein kinase pathway, the mitogen activated protein kinase (MAPK) pathway. Xenon treatment resulted in increased abundance of phosphorylated MAPK (MAPK-p) in injury models of hypoxia-ischemia (Table 6). There was no change in MAPK-p expression when xenon was given in the absence of, or before, injury. This suggests that the observed increase in MAPK-p expression depends on the cellular response to injury, in addition to xenon exposure.

#### 5.2.3. Altering gene transcription

Both the PI3K/Akt and the MAPK pathways converge on cAMP response element binding protein (CREB) and activate the transcription factor by phosphorylation. CREB plays an essential role in neurodevelopment and neural plasticity and has been identified as a potential target for neuroprotective therapies (Sakamoto et al., 2011).

Each study that reported the expression of the phosphorylated, activated form of CREB (CREB-p) identified an increase in the xenon exposure group (Table 6). Increased expression of CREB-p was identified in brain and spinal cord studies, in pre- and post-injury xenon exposure studies and the effect was dose-dependent.

CREB regulates the expression of a number of proteins that promote survival and whose expression is increased following xenon exposure, including Bcl-2 (Meller et al., 2005) and neurotrophic factors such as BDNF (Sakamoto et al., 2011). This suggests that CREB is a significant nexus for xenon action, either as a result of upstream activation, via the PI3K/Akt or MAPK pathway, or a direct interaction between CREB and xenon.

Xenon was also reported to increase expression of the transcription factor HIF1α. In normoxic conditions, HIF1α is generally degraded in the cytoplasm and does not alter gene transcription. However, when activated by the PI3K/Akt pathway, HIF1α can translocate to the nucleus, where it combines with HIF1β to promote the transcription of multiple pro-survival genes (Zhang et al., 2018). HIF1α activation has previously been reported to be responsible for xenon induced nephroprotection against hypoxic-ischemic injury (Ma et al., 2009) and might also play a role in neuroprotection.

Multiple changes in the transcription of mRNA following xenon exposure were identified utilizing suppression subtractive hybridization and multiplex mRNA quantification techniques (Table 7). Whilst these techniques identified altered mRNA transcription for potential mediators of neuroprotection, they also reported altered mRNA transcription for many proteins deemed unrelated to neuroprotection. Whilst these techniques have the advantage of screening a large number of genes to identify alterations in gene transcription, the significance of these transcripts to neuroprotection requires confirmation with *a priori* studies.

**Table 7:** Messenger RNA summary.

**References**	**Method quantification**	**mRNA**	**Tissue**	**Species**	**Injury model**	**Route xenon**	**Xenon dose**	**Xenon duration**	**Direction of effect**
Cattano et al. (2008)	RT PCR	Akt	Brain	Rat	No	Inhaled	75%	2 h	**↓**
Cattano et al. (2008)	RT PCR	JNKK1	Brain	Rat	No	Inhaled	75%	2 h	**↑**
Cattano et al. (2008)	RT PCR	ADNP	Brain	Rat	No	Inhaled	75%	2 h	**↑**
Valleggi et al. (2008)	RT PCR	PI5P4Kβ	Brain	Rat	No	Inhaled	75%	2 h	**↑**
Valleggi et al. (2008)	RT PCR	Prothymosin α	Brain	Rat	No	Inhaled	75%	2 h	**↑**
Valleggi et al. (2008)	RT PCR	Rab14	Brain	Rat	No	Inhaled	75%	2 h	**↑**
Valleggi et al. (2008)	RT PCR	SAP 102	Brain	Rat	No	Inhaled	75%	2 h	**↑**
Valleggi et al. (2008)	RT PCR	Similar to DNER	Brain	Rat	No	Inhaled	75%	2 h	**↑**
Metaxa et al. (2014)	RT PCR	MMP-9	Brain	Rats	Yes	Inhaled	50%	45 min	**↓**
Zhao et al. (2018)	RT PCR	CLIC-4	Brain	Rats	Yes	Inhaled	50%	3 h	**↓**
Filev et al. (2021)	Multiplex	Hmox1	Brain	Rat	Yes	Inhaled	75%	1 h	**↑**
Filev et al. (2021)	Multiplex	Irf1	Brain	Rat	Yes	Inhaled	75%	1 h	**↑**
Filev et al. (2021)	Multiplex	Myd88	Brain	Rat	Yes	Inhaled	75%	1 h	**↑**
Filev et al. (2021)	Multiplex	S100A8	Brain	Rat	Yes	Inhaled	75%	1 h	**↑**
Filev et al. (2021)	Multiplex	Tlr2	Brain	Rat	Yes	Inhaled	75%	1 h	**↑**

Direction of effect (achieved statistical significance): ↑–Increased, ↓–Reduced, ↔–No change. Increases highlighted green. Reductions highlighted red.

#### 5.2.4. Dependence on other intracellular processes

Two studies utilized inhibitors of basic cellular processes, calcium signaling and protein synthesis, to identify those processes that might be essential to xenon neuroprotection. In both cases the neuroprotective properties of xenon were abolished in the presence of these inhibitors (Table 8).

**Table 8:** Injury quantification summary.

**References**	**Cell line/ tissue**	**Species (receptor)**	**Xenon dose**	**Additive**	**Target**	**Outcome reported**	**Xenon protection (no additive)**	**Xenonprotection (additive present)**
Petzelt et al. (2003)	Cortical neurones	Rats	100%	BAPTA	Ca^2+^ signaling	LDH release	+	−
Petzelt et al. (2004)	Phaeochromocytoma cells	Rats	100%	BAPTA	Ca^2+^ signaling	LDH release	+	−
Petzelt et al. (2003)	Cortical neurones	Rats	100%	KN-93	Ca^2+^ signaling	LDH release	+	+
Bantel et al. (2009)	Neuronal-glial coculture	Mouse	75%	Tolbutamide	KATP	Cell viability	+	−
Banks et al. (2010)	Hippocampal brain slice	Mouse	50%	Glycine	NMDA	Normalized injury	+	−
Lavaur et al. (2016)	Cortical/septal cultures	Rats	75%	Glycine	NMDA	Survival rate	+	+
Koziakova et al. (2019)	hippocampal brain slice	Mouse	50%	Glycine	NMDA	Normalized injury	+	−
Harris et al. (2013)	Hippocampal brain slice	Mouse	50%	Glycine	NMDA	Normalized injury	+	−
Ma et al. (2006)	Neuronal culture	Rats	75%	Cyclohexamide	Protein synthesis	LDH release	+	−

+ (green filled box)—xenon provided neuroprotection vs. control condition.

− (red filled box)—xenon did not provide protection vs. control condition.

In both studies, the inhibitors themselves did not induce injury and the abolition of xenon protection suggests that the effect of xenon is dependent on the cellular processes investigated. Both calcium signaling and protein synthesis represent core cellular functions so it is perhaps not surprising that such broad disruptions in function might interfere with xenon neuroprotection.

### 5.3. Limitations of the review

The phenomena of anesthesia and neuroprotection cannot solely be explained by studies at the cellular level and the results of the review need to be considered in the context of studies at other levels of organization within neuroscience, from structural and molecular biology to neural circuit and functional connectivity studies. To provide a comprehensive review, and as a reflection of the expertise within the research team, the focus of the review was maintained on actions at the cellular level.

The review is also limited in its focus to studies of mammalian species. There are several advantages to the study of molecular biology in smaller animals and prokaryotic species, with simpler genomes and faster replication times. These studies unquestionably contribute to our understanding of how general anesthetic agents work (Forman, 2006). However, findings in these simpler systems require replication in mammalian systems to confirm the relevance of the findings to humans.

The “protein hypothesis” of general anesthetic action has gained ascendancy in recent decades, supplanting the “lipid hypothesis” as the primary mechanistic explanation for anesthesia. Whilst the review is not intended to endorse one paradigm over the other, in practice, the studies identified by the review largely describe the interaction of xenon with proteins. The review sought to identify molecules that xenon might interact with at the cellular level and terms such as “phospholipid” or “lipid bilayer” were not specified in the search.

In defense of the review findings, disruption of the lipid bilayer itself is unlikely to account for the phenomenon of general anesthesia. More likely it is a pathway by which membrane bound proteins or cell signaling cascades are activated. A recent investigation in drosophila identified that disruption of the lipid raft and activation of phospholipases were initial events that led to the activation of P2K channels (Pavel et al., 2020). These processes, the activity of cell bound channels and intracellular signaling, are well represented within the review.

## 6. Conclusions

Since the first report of xenon's ability to antagonize NMDAR, xenon has been assigned a place alongside the “NMDA antagonist” anesthetic agents ketamine and nitrous oxide. Whilst xenon does antagonize excitatory glutamatergic transmission, its specific action at the glycine binding site of NMDAR distinguishes it from ketamine and nitrous oxide, and is reflected in distinct behavioral and electrophysiological characteristics.

The effects of xenon on cortical activity, as reflected in the electroencephalogram, bear a greater resemblance to anesthetic agents with activity at GABAR than the NMDAR antagonists. In the absence of significant activity at GABAR, xenon's electrophysiological features might reflect its action at thalamic molecular targets such as HCN2 and the NR2C NMDAR subtype.

Xenon has unique potential as a neuroprotectant due to its wide range of molecular targets and tolerability. Xenon shares neuroprotective mechanisms with both NMDAR antagonists and volatile anesthetic agents. It also has unique activity at cell membrane targets such as KATP. The effects of xenon are not limited to reducing excitability via cell membrane targets as xenon also influences protein signaling cascades and gene transcription to promote cell survival.

Whilst xenon was administered at or above anesthetic doses in many studies, subanesthetic doses were effective in altering the activity of many cell membrane bound and intracellular proteins. Utilizing subanesthetic concentrations might improve the accessibility and tolerability of xenon. Outside the operating room, utilizing subanesthetic doses can avoid excessive sedation whilst also reducing the costs of administration.

The description of xenon as an “ideal anesthetic” does not account for its relative scarcity, high cost and requirement for specialist equipment for delivery. However, xenon does possess unique beneficial characteristics when compared to its contemporaries. Understanding its mechanism of action might aid the development of newer anesthetic and neuroprotective agents which can provide similar benefits to xenon, whilst being more accessible to clinicians.

## Data availability statement

The original contributions presented in the study are included in the article/Supplementary material, further inquiries can be directed to the corresponding author.

## Author contributions

SM: study design, data collection, and manuscript preparation. DM and LO'B: data collection and manuscript review. FF, BS, LE, and DS: study design and manuscript review. All authors contributed to the article and approved the submitted version.
