# Supplementary material for: The cellular mechanisms associated with the anesthetic and neuroprotective properties of xenon: a systematic review of the preclinical literature
Source: Front Neurosci. 2023 Jul 14;17:1225191. doi: 10.3389/fnins.2023.1225191 (PMC10380949; doi:10.3389/fnins.2023.1225191)
Supplement: Supplementary file 1 [file Data_Sheet_1.docx]

**Supplementary materials – search strategy for:**

The cellular mechanisms associated with the anesthetic and neuroprotective properties of xenon: a systematic review of the pre-clinical literature

**Search terms entered for Ovid MEDLINE(R) ALL 1946 to May 26, 2022**

1. exp Xenon/

2. xenon.ti,ab.

3. 1 or 2

4. exp Anesthetics/

5. exp Anesthesia/

6. an?esthe*.ti,ab.

7. exp Neuroprotection/

8. exp Neuroprotective Agents/

9. (neuro-protect* or neuroprotect* or neuro-toxic* or neurotoxic* or neuro-apoptosis or neuroapoptosis or organo-protect* or organoprotect* or pre-condition* or precondition* or post-condition* or postcondition* or neuro-inflamm* or neuroinflamm* or neuronal injury or brain injury).ti,ab.

10. 4 or 5 or 6 or 7 or 8 or 9

11. exp signal transduction/ or exp second messenger systems/ or exp synaptic transmission/

12. exp Synaptic Potentials/

13. exp membrane transport proteins/ or exp ion channels/ or exp calcium channels/ or exp chloride channels/ or exp cysteine loop ligand-gated ion channel receptors/ or exp receptors, ionotropic glutamate/ or exp potassium channels/ or exp sodium channels/ or exp ion pumps/

14. exp Receptors, Cell Surface/

15. Calcium/me [Metabolism]

16. exp gene expression/ or exp Transcription, genetic/ or exp gene expression regulation/

17. (mechanism of action or molecular action* or molecular pharmacology or molecular mechanism* or neurophysiological mechanism* or synap* or receptor* or channel* or membrane protein* or signal transduction or second messenger* or signal?ing or calcium or transcription or expression or up?regulat*).ti,ab.

18. 11 or 12 or 13 or 14 or 15 or 16 or 17

19. 3 and 10 and 18

**Additional eligibility criteria**

**Proteins/molecules considered potential targets for xenon (included)**

All protein kinases

All transcription factors

Members of mitochondrial apoptosis cascade

**Proteins/molecules not considered potential targets of xenon (excluded)**

c-Fos

Glial fibrillary acidic protein (GFAP)

Lactate dehydrogenase (LDH)

Terminal deoxynucleotidyl transferase dUTP nick end labeling (TUNEL)
