# Supplementary material for: The cellular mechanisms associated with the anesthetic and neuroprotective properties of xenon: a systematic review of the preclinical literature
Source: Front Neurosci. 2023 Jul 14;17:1225191. doi: 10.3389/fnins.2023.1225191 (PMC10380949; doi:10.3389/fnins.2023.1225191)
Supplement: Supplementary file 2 [file Table_1.docx]

**OHAT risk of bias assessment – response matrix for:**

The cellular mechanisms associated with the anesthetic and neuroprotective properties of xenon: a systematic review of the preclinical literature

| Author | Year | 1. Randomization | 2. Allocation concealment | 3. Experimental conditions | 4. Blinding (during study) | 5. Complete outcome data | 6. Exposure characterisation | 7. Outcome assessment | 8. Outcome reporting | 9. Other |
| --- | --- | --- | --- | --- | --- | --- | --- | --- | --- | --- |
| Armstrong | 2012 | NA | NA | DL | PL | PH | PH | DL | PL | DL |
| Banks | 2010 | NA | NA | DL | PL | DL | PH | DL | DL | DL |
| Bantel | 2009 | NA | NA | DL | PL | DL | PH | DL | PL | DL |
| Bantel | 2010 | NA | NA | DL | PL | PL | PH | DL | PL | DL |
| Baufreton | 2018 | NA | NA | DL | PH | DL | PH | PH | PL | PH |
| Cattano | 2011 | PL | PH | DL | PH | DL | DL | DL | PH | PL |
| Cattano | 2008 | PL | PH | DL | PL | DL | DL | DL | PH | PH |
| Dandekar | 2018 | PL | PH | DL | PH | PL | DL | DL | DL | PH |
| Daniels | 1998 | NA | NA | DL | PL | DL | DL | DL | DL | PH |
| David | 2006 | NA | NA | DL | PL | PL | PH | DL | DL | DL |
| David | 2008 | NA | NA | DL | PL | PL | DL | DL | PL | DL |
| David | 2003 | NA | NA | DL | PL | PL | PH | DL | DL | DL |
| DeSousa | 2000 | NA | NA | DL | PL | PL | PL | DL | DL | DL |
| Dickinson | 2007 | NA | NA | DL | PL | PL | PH | DL | DL | DL |
| Dinse | 2005 | NA | NA | DL | PL | PL | DL | PL | PL | PL |
| Eger | 2006 | PL | PL | DL | PL | PL | DL | PH | DL | DL |
| Fahlenkamp | 2011 | NA | NA | DL | PL | DL | PH | DL | DL | PH |
| Fan | 2021 | DL | PH | DL | PH | PL | PH | DL | DL | DL |
| Filev | 2021 | PL | PL | PL | DL | PH | DL | DL | PH | DL |
| Franks | 1995 | NA | NA | DL | PL | PL | DL | DL | PH | PL |
| Franks | 1998 | NA | NA | DL | PL | PL | PL | DL | DL | DL |
| Fukuda | 2002 | PH | PH | PH | DL | DL | DL | DL | DL | DL |
| Georgiev | 2010 | PH | PH | DL | PH | PL | PH | DL | PL | PL |
| Gruss | 2004 | NA | NA | DL | PL | PL | DL | DL | DL | DL |
| Hapfelmeier | 2000 | NA | NA | DL | PL | PL | DL | DL | PL | DL |
| Harris | 2013 | NA | NA | PH | PL | PL | PH | DL | PL | PL |
| Haseneder | 2008 | NA | NA | DL | PL | PL | DL | DL | PL | PL |
| Haseneder | 2009a | NA | NA | DL | PL | PL | DL | DL | DL | PL |
| Haseneder | 2009b | NA | NA | DL | PL | PH | DL | DL | PL | PL |
| Horn | 1995 | NA | NA | DL | PL | PL | PL | DL | DL | DL |
| Jin | 2021 | PH | PH | DL | PH | DL | DL | DL | DL | DL |
| Koziakova | 2019 | NA | NA | DL | PL | PL | PL | DL | DL | DL |
| Kratzer | 2012 | NA | NA | PH | PL | PL | DL | DL | DL | DL |
| Kubota | 2020 | NA | NA | DL | PL | PL | PH | DL | DL | PL |
| Kuzovlev | 2021 | PH | PH | DL | PH | DL | PH | PH | DL | PH |
| Lavaur | 2016 | NA | NA | DL | PL | DL | PH | DL | DL | DL |
| Lehmke | 2018 | NA | NA | DL | PL | DL | DL | DL | DL | DL |
| Limatola | 2010 | PL | PH | DL | PL | DL | PH | DL | DL | DL |
| Liu | 2016 | DL | PH | DL | PL | DL | PH | DL | PL | DL |
| Luo | 2008 | PH | PH | DL | PL | DL | PH | DL | DL | DL |
| Ma | 2006 | PH | PH | DL | PL | DL | DL | DL | DL | PL |
| Ma | 2007 | PH | PH | DL | PL | PH | DL | DL | DL | DL |
| Mattusch | 2015 | NA | NA | PL | PL | PL | DL | DL | DL | PL |
| Metaxa | 2014 | PL | PH | PH | PH | DL | DL | DL | DL | PL |
| Neukirchen | 2012 | NA | NA | DL | PL | DL | DL | DL | DL | DL |
| Nonaka | 2019 | NA | NA | DL | PL | DL | PH | DL | DL | DL |
| Ogata | 2006 | NA | NA | DL | PL | PL | DL | DL | DL | DL |
| Peng | 2013 | PL | DL | PH | PL | DL | PH | DL | DL | PL |
| Petzelt | 2003 | NA | NA | DL | PL | DL | DL | DL | DL | DL |
| Petzelt | 2004 | NA | NA | DL | PL | DL | PH | PH | DL | PL |
| Plested | 2004 | NA | NA | DL | PL | PL | DL | DL | PL | PL |
| Shu | 2010 | PH | PH | DL | PL | PL | DL | DL | DL | PL |
| Singh | 1995 | NA | NA | DL | PL | DL | PL | PH | DL | DL |
| Solt | 2006 | NA | NA | DL | PL | DL | PH | DL | DL | PH |
| Suzuki | 2002 | NA | NA | DL | PL | DL | DL | DL | DL | DL |
| Suzuki | 2003 | NA | NA | DL | PL | PL | DL | DL | DL | DL |
| Vallegi | 2008 | PL | PH | DL | PL | DL | DL | PH | DL | PL |
| Vizcaychipi | 2011 | PH | PH | PL | PL | PL | DL | PH | DL | DL |
| Weigt | 2003 | NA | NA | DL | PL | PL | DL | DL | DL | DL |
| Weigt | 2008 | NA | NA | DL | PL | DL | PH | PH | PL | PL |
| Weigt | 2009a | NA | NA | DL | PL | PL | DL | DL | PL | PL |
| Weigt | 2009b | NA | NA | DL | PL | PL | DL | DL | DL | DL |
| White | 2005 | NA | NA | DL | PL | DL | PL | DL | DL | PL |
| White | 2011 | NA | NA | DL | PL | DL | PH | DL | PL | DL |
| Yamakura | 2000 | NA | NA | DL | PL | PL | DL | DL | DL | PL |
| Yamamoto | 2012 | NA | NA | DL | PL | DL | PH | DL | DL | DL |
| Yang | 2014 | DL | DL | DL | DL | DL | DL | DL | DL | DL |
| Zhao | 2018 | PL | DL | DL | PL | PL | PH | DL | DL | DL |
| Zhuang | 2012 | PL | DL | DL | PL | PL | PH | DL | DL | DL |

DL – Definitely low, PL – Probably low, PH – Probably high, NA – not applicable

OHAT Handbook (https://ntp.niehs.nih.gov/ntp/ohat/pubs/handbookmarch2019_508.pdf)

**OHAT risk of bias assessment – Summary of responses**
